# Clusters in craniofacial microsomia and microtia according to facial morphology and craniofacial anomalies

**DOI:** 10.1007/s00431-026-06973-9

**Published:** 2026-04-24

**Authors:** Elsa M. Ronde, Guido A. de Jong, Jitske W. Nolte, Marloes E. L. Nienhuijs, Neil W. Bulstrode, Thomas J. J. Maal, Alfred G. Becking, Corstiaan C. Breugem, The 3DCFMM research consortium, A. G. Becking, J. Bolks, C. C. Breugem, N. W. Bulstrode, D. Dunaway, F. A. Ebbens, G. A. de Jong, F. H. Kruisinga, Christiane Landwehr, T. J. J. Maal, M. E. L. Nienhuijs, J. W. Nolte, E. M. Ongkosuwito, E. M. Ronde, B. J. van Royen, S. Schievano, A. Stadhouder, M. Tjaberinga

**Affiliations:** 1 Department of Plastic, Reconstructive and Hand Surgery, Amsterdam UMC, University of Amsterdam, Meibergdreef 9, Amsterdam, 1105AZ the Netherlands; 2 Department of Oral and Maxillofacial Surgery, Amsterdam UMC, University of Amsterdam and Academic Centre Dentistry Amsterdam, Amsterdam, the Netherlands; 3 Amsterdam Reproduction and Development Research Institute, Amsterdam, the Netherlands; 4 Amsterdam UMC Expert Center for Cleft, Craniofacial and Airway Disorders, Amsterdam, the Netherlands; 5 Department of Oral and Maxillofacial Surgery, Radboudumc, Nijmegen, the Netherlands; 6 Radboudumc 3D Lab, Radboudumc, Nijmegen, the Netherlands; 7 Department of Plastic Surgery, Great Ormond Street Hospital, London, United Kingdom; 8 UCL Great Ormond Street Institute of Child Health, London, United Kingdom

**Keywords:** Craniofacial microsomia, Stereophotogrammetry, Ocular anomalies, Aural atresia, Clefting, Velopharyngeal dysfunction

## Abstract

Associated craniofacial malformations such as ocular anomalies and macrostomia are frequently seen in craniofacial microsomia (CFM). This study assessed associations between objectively measured facial morphology and clinically observed craniofacial malformations in patients with CFM in order to identify clinically significant patient clusters based on these characteristics for preferential screening purposes. In this multicenter cross-sectional cohort study, facial morphology was assessed from three-dimensional (3D) photographs using asymmetry index (ASI) and facial signature (FS) scores, as well as a principal components (PC) analysis of FS scores. Differences in ASI, FS and PC scores were calculated for patients with and without craniofacial anomalies. A clustering analysis was applied to identify patient clusters based on facial morphology and the presence of craniofacial anomalies. A total of 179 patients were included, and significantly higher ASI and FS scores were found in patients with ocular anomalies, skin adnexa-related anomalies, clefting, inner ear anomalies, speech- and language difficulties and vision difficulties compared to patients without these anomalies or difficulties. Two clusters were identified, with significant differences in PC scores, craniofacial anomalies, as well as speech- and language- and vision difficulties.

*Conclusion*: Patients with ocular anomalies, skin adnexa-related anomalies, clefting and aural atresia, or anomalies in two or more of these groups had more facial hypoplasia, while patients with anomalies in two or fewer of these groups had less facial hypoplasia. 3D photography may be a promising tool to identify patients that should be screened for these concurrent craniofacial anomalies.

**Table:** 

**What Is Known:** *• CFM is a hetergeneous condition and patients may have various craniofacial anomalies.* • *3D photography can be used to measure facial shape in patients with CFM.* **What Is New:** *• Patients with more facial hypoplasia measured using 3D photography frequently have more associated craniofacialanomalies.* *• 3D photography could be a promising tool for identifying patients who should be preferentially screened forassociated anomalies.*

**Supplementary information:**

The online version contains supplementary material available at 10.1007/s00431-026-06973-9.

## Introduction

Craniofacial microsomia (CFM) is a congenital craniofacial condition with an estimated prevalence ranging from 1 in 5500 to 1 in 26,000 live births [1, 2]. This large range in estimated prevalence can likely be attributed to the range in phenotypic variability, which arguably ranges from isolated microtia to bilateral hypoplasia of skeletal and soft-tissue structures of the face, and the ears [3–5]. CFM is also frequently associated with other craniofacial anomalies such as epibulbar dermoids and skin tags, as well as cleft lip and/or palate (CL/P) and macrostomia [2]. Individuals with CFM are also at an increased risk for speech- and language difficulties, notably including velopharyngeal dysfunction (VPD) [6] even in the absence of CL/P [7].

Although these anomalies are frequently seen, there are no clear indications on which patients should be preferentially screened for these anomalies and difficulties after birth. Ocular anomalies, CL/P and macrostomia have been reported to occur more frequently in bilaterally affected patients [8, 9]. However, the assessment of the severity of facial involvement in bilaterally affected patients is limited by the currently used Orbit, Mandible, Ear, Nerve and Soft tissue (OMENS) classification tool, which depends on the contralateral side of the face for reference in assessing orbital and soft tissue hypoplasia in particular [10]. Several studies have also reported bilateral morphological changes even in seemingly unilaterally affected patients [5, 11, 12], which further complicates the distinction between unilaterally and bilaterally affected patients and emphasizes the need for comprehensive facial morphological assessment instead.

Digital stereophotogrammetry (3D photography) can be used to objectively assess facial morphology of patients with CFM in a clinically relevant way [12]. The aim of the current study was to assess associations between facial morphology, evaluated using 3D photography, and craniofacial malformations, as well as hearing-, vision- and speech- and language difficulties in patients with CFM and microtia. This study also aimed to identify clusters of patients based on the objective 3D assessment of facial morphology and the presence of craniofacial anomalies in order to clarify which patients may benefit from screening for associated anomalies.

## Methods

Patients with CFM according to the International Consortium for Health Outcomes Measurement [13] criteria or isolated microtia with 3D photographs of good quality (i.e. neutral facial expression, no movement artefacts and no large data holes in the facial region) were included from the Amsterdam UMC and Radboudumc in the Netherlands, and the Great Ormond Street Hospital for Children in the United Kingdom. Patients who had undergone significant craniofacial surgery, with the exception of cleft lip repair or primary cleft palate repair, as well as patients with confirmed syndromes (e.g. Treacher Collins) were excluded. Patients with ‘Goldenhar syndrome’ were considered to have CFM [13].

Data was retrospectively collected on patient characteristics (e.g. year of birth and sex), as well as CFM-related characteristics using the Phenotypic Assessment Tool for Craniofacial Microsomia (PAT-CFM) global assessment, which corresponds to the OMENS score, as well as the PAT-CFM detailed assessment from patient files and available images. Clinically bilateral CFM was defined as scores of > 0 in at least one subscale of the OMENS on both sides of the face. The following data on craniofacial anomalies were registered: ocular anomalies (colobomata, strabismus and epibulbar anomalies), skin-adnexa related anomalies (skin tags and skin pits), clefting (macrostomia, cleft lip and/or cleft palate [CL/P], and submucous clefting), nerve weakness (facial nerve weakness and dynamic uvula deviation), aural atresia, middle ear anomalies and inner ear anomalies. Middle- and inner ear anomalies were defined based on computed tomography or magnetic resonance imaging. Any other craniofacial anomalies mentioned in patient files were also registered and grouped within the aforementioned groups (e.g. bifid uvula grouped under ‘clefting’). Data on the presence and type of any hearing, vision and speech- and language difficulties were also registered. Speech- and language difficulties were defined as any mention of speech- and language-related problems requiring treatment by a speech- and language therapist. VPD was defined by at least a description of hypernasality or nasal air emission in the patient files [14]. If no mention of the specified anomaly, or hearing, speech- and language, or vision difficulties were reported in patient files, these were considered not present.

This study adhered to local institutional review board approvals or exemptions, as well as the Strengthening the Reporting of Observational studies in Epidemiology (STROBE) checklist (Online Resource 1) [15].

### Facial morphology analyses

The facial morphology analysis has been validated and described in detail previously [12]. Briefly, all 3D photographs were standardized using 3DMedX version 1.2.28.1 (Radboudumc 3D Lab, Nijmegen, Netherlands) and the validated Meshmonk toolbox to consist of 7160 bilaterally and anatomically comparable datapoints [16, 17]. Patients’ facial morphology was objectively assessed using two measures: the asymmetry index (ASI), a measure for the degree of bilateral symmetry, measured for clinically unilateral patients only, and facial signature (FS) scores for all patients. FS scores are a measure for the degree of deviation from an age- and sex-adjusted normative population mean, derived from a model of more than 5000 3D images [18]. Both were summarized for the whole face, as well as 10 regions depicting the upper- and lower halves of the face and the midface. These facial regions were selected due to their clinical relevance in the CFM population [19, 20]. A principal components analysis (PCA) was initially performed on the FS scores of individuals without craniofacial conditions (from the Large Scale Facial Model dataset [21] and the Radboudumc 3D Lab dataset) and subsequently applied to the patient population. Then, a logistic regression of these principal components (PCs) yielded nine PCs which significantly distinguished patients from the individuals without craniofacial conditions (Online Resource 2). The resulting PCs each represented a pattern in facial shape where the shape pattern of these nine significant PCs involved both symmetrical and asymmetrical changes in the orbits, mandible and cheeks (Online Resource 2). In the previous validation study, the regression model of these nine PCs was correlated to clinical OMENS scores indicating that it represented clinically meaningful facial shape patterns to CFM [12]. These nine PCs were used in the current study to assess associations between these significant patterns in facial morphology, as well as clinically identified craniofacial malformations and speech- and language-, hearing- and vision difficulties.

### Statistical analyses

All analyses were performed using R version 4.2.1 (R Core Team, Vienna, Austria) via the RStudio interface [22]. Data was summarized as number (%), median (interquartile range) or mean (standard deviation), depending on variable type and distribution. Differences in ASI, FS and PC scores were calculated for patients with and without anomalies in the aforementioned groups, as well as speech- and language, hearing and vision difficulties, using the Mann–Whitney *U* test for ASI and FS scores and Student’s *t*-tests for PC scores.

A clustering analysis was performed in order to identify potential patient clusters based on a combination of patients’ facial morphology and anomalies in one or more of the following groups: ocular anomalies, skin adnexa-related anomalies, clefting and aural atresia. These groups of anomalies were selected as these are clinically identifiable without radiographic imaging and were easily extractable from patient files and photographs. They were also treated as binary variables (e.g. patients with at least one ocular anomaly vs. patients with no reported ocular anomalies). Clustering was performed with the nine PCs and the aforementioned groups of anomalies as input variables using the partitioning around medoids algorithm [23], and the Gower distance, which is suitable for mixed data [24]. The number of clusters was determined using cluster validation measures with the clValid package in R and visualized using multidimensional scaling plots. Correlations between the cluster assignments and the OMENS subscales were analyzed using the Spearman rank correlation coefficient. Subgroup analyses were performed for patients without clefting with the exception of macrostomia, as well as patients without any clefting at all. A *p* < 0.05 was considered statistically significant. *p*-values for the clustering and subgroup analyses were adjusted for multiple testing using the Benjamini–Hochberg method [25].

## Results

A total of 179 patients were included with a median age of 9 years (interquartile range 5–14 years) at the time of 3D photography. Out of these patients, 25% had ocular anomalies, 56% had skin adnexa-related anomalies, 48% had nerve weakness, 32% had clefting, 82% had aural atresia, and 42% and 18% had middle- and inner ear anomalies, respectively (Table 1). Patients most frequently had anomalies in two (22%), three (25%) or four (22%) of these groups. Strabismus was the most commonly reported ocular anomaly (14% of patients), while skin tags were reported in 51% of patients and facial nerve weakness in 44% of patients. Macrostomia was the most frequently observed form of clefting, reported in 20% of patients. The most frequently reported middle ear malformation was a form of ossicular malformation (36%), while dysplasia of the semicircular canals was the most commonly reported inner ear malformation (11%) (Online Resource 3). Speech- and language difficulties were noted in 49% of patients (Table 1). Speech delay was most frequently reported (20%), followed by VPD (15%) (Online Resource 4). Hearing and vision difficulties were reported in 84% and 27% of patients, respectively (Table 1).

**Table 1:** Summary of craniofacial anomalies and speech- and language, hearing and vision difficulties

	Total no. patients (%), *n* = 179	Side of anomaly		
		*Unilateral (right), n* = *85*	*Unilateral (left), n* = *75*	*Bilateral, n* = *19*
Patient characteristics				
Age	9.0 [5.0–14.0]	10.0 [5.0–15.0]	9.0 [5.0–13.0]	8.0 [4.0–12.0]
Sex, male^1^	98 (55)	49 (58)	43 (57)	6 (32)
Isolated microtia^3^	15 (8)	10 (12)	5 (7)	0
OMENS^1,^^2^				
Orbit, normal size and position	78 (44)	37 (44)	35 (47)	6 (32)
Mandible, 2 A or 2B	57 (32)	23 (27)	26 (35)	8 (42)
Ear, lobule type	72 (40)	35 (41)	31 (41)	6 (32)
Nerve, no facial nerve weakness	65 (35)	38 (45)	23 (31)	4 (21)
Soft tissue, minimal deficiency	86 (46)	46 (54)	33 (44)	7 (37)
Craniofacial anomalies^4^				
Ocular anomalies	44 (25)	12 (7)	17 (10)	15 (8)
Skin adnexa-related anomalies	101 (56)	30 (17)	37 (21)	34 (19)
Nerve weakness	94 (48)	40 (22)	30 (17)	24 (13)
Clefting	57 (32)	16 (9)	12 (7)	29 (16)^5^
Aural atresia	146 (82)	76 (43)	55 (31)	15 (8)
Middle ear anomalies	75 (42)	27 (15)	29 (16)	19 (11)
Inner ear anomalies	33 (18)	8 (5)	12 (7)	13 (7)
Speech, hearing and vision^4^				
Speech and language difficulties	87 (49)	n/a	n/a	n/a
Hearing difficulties	150 (84)	67 (37)	52 (29)	31 (17)
Vision difficulties	48 (27)	6 (3)	11 (6)	30 (17)

^1^Proportions of characteristics for unilateral and bilateral patients relate to the total number of unilateral and bilateral patients

^2^For bilaterally affected patients, scores of the more severely affected side are described

^3^According to the OMENS classification

^4^Proportions of characteristics relate to the total number of the particular anomaly or difficulty

^5^Central clefting categorized under bilateral. Combinations including central clefting categorized as bilateral

### ASI, FS and PC scores

Significantly higher ASI and FS scores were found for patients with ocular anomalies, skin adnexa-related anomalies, clefting, inner ear anomalies, speech- and language difficulties and vision difficulties in several facial regions compared to patients without these anomalies or difficulties (Online Resources 5 and 6). With regard to the principal component scores, significant differences were mainly found between patients with skin adnexa-related anomalies or clefting and those without (Online Resource 7).

### Clustering analysis

**Fig. 1:**
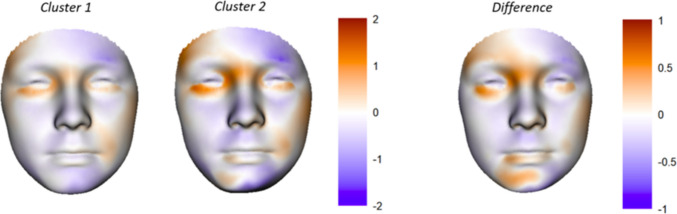
Visualization of a mean patient in cluster 1 (left) and cluster 2 (middle) according to the cluster analysis and visualized using the PCA model. The difference between clusters is visualized on the right. The corresponding FS scores are overlaid on the images, according to the color scale where orange represents positive FS scores and purple represents negative FS scores

Two clusters were identified (Online Resources 8), with significant differences in PC scores, craniofacial anomalies, as well as speech- and language-, and vision difficulties, where patients in cluster 2 were more frequently clinically diagnosed with ocular anomalies, skin adnexa-related anomalies, clefting (including macrostomia) and aural atresia, or anomalies in two or more of these groups (Table 2). Patients in cluster two also had a more severe facial phenotype (Fig. 1), where differences between clusters were mainly characterized by an increased degree of (bilateral) orbital involvement and chin deviation.

**Table 2:** Differences in PC scores, craniofacial anomalies, and speech- and language**,** hearing and vision difficulties between clusters 1 and 2

	Cluster 1, *n* = 121^1^	Cluster 2, *n* = 58^1^	*p-value*^*3*^
Bilateral involvement according to OMENS	9 (7.4%)	10 (17%)	0.074
Principal component			
PC13	− 4.73 (12.31)	− 5.49 (11.66)	0.7
PC14	− 8.96 (16.62)	− 7.20 (20.30)	0.6
PC15	5.67 (15.62)	12.00 (20.76)	0.046
PC19	− 1.80 (11.08)	− 3.33 (16.13)	0.6
PC20	− 14.19 (14.15)	− 25.92 (24.15)	< 0.001
PC23	− 6.85 (9.17)	− 10.05 (11.92)	0.075
PC30	8.42 (10.46)	15.48 (12.70)	< 0.001
PC33	1.90 (6.20)	2.47 (7.21)	0.6
PC34	− 0.89 (5.81)	− 2.84 (6.14)	0.068
Craniofacial anomalies			
Ocular anomalies	9 (7.4%)	35 (60%)	< 0.001
Skin adnexa-related anomalies	49 (40%)	52 (90%)	< 0.001
Nerve weakness	65 (54%)	29 (50%)	0.7
Clefting	9 (7.4%)	48 (83%)	< 0.001
Aural atresia	105 (87%)	41 (71%)	0.02
Middle ear anomalies	54 (45%)	21 (36%)	0.4
Inner ear anomalies	17 (14%)	16 (28%)	0.052
No. of anomalies in different groups^4^			< 0.001
0	8 (6.6%)	0 (0%)	
1	54 (45%)	0 (0%)	
2	59 (49%)	11 (19%)	
3	0 (0%)	34 (59%)	
4	0 (0%)	13 (22%)	
Speech, hearing and vision			
Speech and language difficulties	50 (41%)	37 (64%)	0.012
Velopharyngeal dysfunction	11 (9.1%)	15 (26%)	0.008
Hearing difficulties	103 (85%)	47 (81%)	0.6
Vision difficulties	26 (21%)	22 (38%)	0.04

^1^Mean (SD); number (%)

^2^Two sample *t*-test; Pearson’s Chi-squared test. Adjusted according to the Benjamini–Hochberg method

^3^Grouped according to the presence of anomalies in the following groups: ocular anomalies, skin adnexa-related anomalies, clefting and aural atresia

**Fig. 2:**
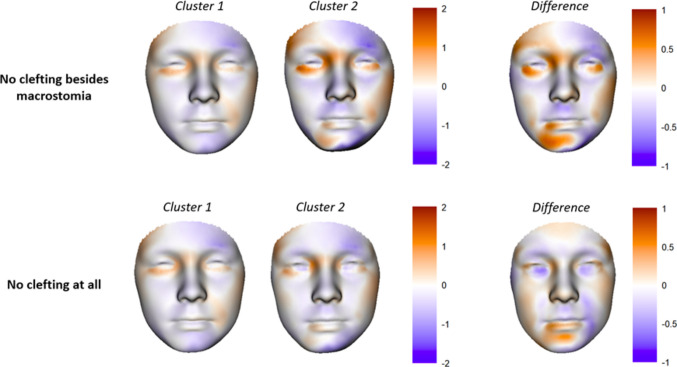
Visualization of a mean patient without cleft lip- and/or palate (CL/P), submucous clefting (SM) or a bifid uvula in clusters 1 and 2 (left) and a mean patient without any clefting in clusters 1 and 2 (middle). The difference between clusters is visualized on the right. The corresponding FS scores are overlaid on the images, according to the color scale where orange represents positive FS scores and purple represents negative FS scores

In the subgroup analyses, significant differences were still seen in several PCs and the number of different craniofacial anomalies for the subgroup including macrostomia, and mainly the number of different craniofacial anomalies in the subgroup excluding all types of clefting (Table 3). The cluster of patients with more associated craniofacial anomalies (cluster 2) had a more severe facial phenotype in both subgroups, although this difference was less pronounced in the subgroup without any clefting (Fig. 2). Differences in facial morphology between clusters was characterized by the degree of chin deviation in both subgroups, as well as the extent of (bilateral) orbital involvement in the macrostomia subgroup.

**Table 3:** Principal component scores, craniofacial anomalies, and speech and language-, hearing- and vision difficulties per cluster for the subgroup analyses

	No clefting besides macrostomia			No clefting		
	Cluster 1, *n* = 116^1^	Cluster 2, *n* = 32^1^	*p*-value^2^	Cluster 1, *n* = 112^1^	Cluster 2, *n* = 10^1^	*p*-value^2^
Bilateral involvement according to OMENS	8 (6.9%)	2 (6.3%)	> 0.9	8 (7.1%)	2 (20%)	0.4
Principal component						
PC13	− 4.90 (12.39)	− 3.41 (12.01)	0.7	− 4.74 (12.54)	− 0.94 (11.56)	0.6
PC14	− 8.80 (16.35)	− 3.19 (21.59)	0.2	− 8.61 (16.20)	3.68 (12.99)	0.063
PC15	5.61 (15.57)	16.50 (17.38)	0.002	5.19 (14.59)	8.61 (18.05)	> 0.9
PC19	− 1.76 (11.25)	1.06 (13.61)	0.4	− 1.80 (10.67)	− 2.46 (11.30)	> 0.9
PC20	− 14.25 (14.21)	− 29.27 (24.51)	< 0.001	− 13.46 (13.68)	− 15.90 (22.48)	> 0.9
PC23	− 6.83 (9.25)	− 10.96 (10.71)	0.06	− 6.57 (9.27)	− 7.23 (13.49)	> 0.9
PC30	8.36 (10.26)	17.09 (13.41)	< 0.001	7.93 (10.09)	13.16 (11.87)	0.4
PC33	1.98 (6.00)	1.63 (5.86)	0.8	2.04 (5.97)	1.25 (4.86)	> 0.9
PC34	− 0.76 (5.80)	− 4.21 (4.35)	0.005	− 0.54 (5.64)	− 5.73 (4.71)	0.046
Craniofacial anomalies						
Ocular anomalies	9 (7.8%)	21 (66%)	< 0.001	9 (8.0%)	9 (90%)	< 0.001
Skin adnexa-related anomalies	49 (42%)	31 (97%)	< 0.001	49 (44%)	10 (100%)	0.004
Nerve weakness	60 (52%)	16 (50%)	0.9	59 (53%)	6 (60%)	> 0.9
Clefting	4 (3.4%)	22 (69%)	< 0.001	n/a	n/a	n/a
Aural atresia	100 (86%)	22 (69%)	0.046	96 (86%)	9 (90%)	> 0.9
Middle ear anomalies	51 (44%)	6 (19%)	0.02	49 (44%)	2 (20%)	0.4
Inner ear anomalies	16 (14%)	8 (25%)	0.2	15 (13%)	2 (20%)	> 0.9
No. of anomalies in different groups^3^			< 0.001			< 0.001
0	8 (6.9%)	0 (0%)		8 (7.1%)	0 (0%)	
1	54 (47%)	0 (0%)		54 (48%)	0 (0%)	
2	54 (47%)	6 (19%)		50 (45%)	2 (20%)	
3	0 (0%)	20 (63%)		0 (0%)	8 (80%)	
4	0 (0%)	6 (19%)		n/a	n/a	
Speech, hearing and vision						
Speech and language difficulties	45 (39%)	14 (44%)	0.7	42 (38%)	3 (30%)	> 0.9
Velopharyngeal dysfunction	6 (5.2%)	2 (6.3%)	0.8	6 (5.4%)	0 (0)	> 0.9
Hearing difficulties	98 (84%)	25 (78%)	0.6	94 (84%)	9 (90%)	> 0.9
Vision difficulties	25 (22%)	11 (34%)	0.2	25 (22%)	6 (60%)	0.063

^1^Mean (SD); number (%)

^2^Two sample *t*-test; Pearson’s Chi-squared test; Fisher’s exact test. P-values adjusted according to the Benjamini–Hochberg method

^3^Grouped according to the presence of anomalies in the following groups: ocular anomalies, skin adnexa-related anomalies, clefting and aural atresia

Cluster assignments were significantly positively correlated to the Pruzansky-Kaban score and the soft tissue subscale, as well as negatively to the ear subscale of the OMENS classification. In the subgroup analyses, cluster assignments were significantly positively correlated with the Pruzansky-Kaban score and the soft tissue subscale of the OMENS for the macrostomia subgroup (Table 4).

**Table 4:** Correlation between cluster assignments and OMENS subscales for the primary and both subgroup analyses

		Orbit	Mandible^1^	Ear	Nerve	Soft tissue
All patients,* n* = *179*	*No. of patients*	177	132	173	131	176
	*Correlation coefficient*^*2*^	0.101	0.350	− 0.337	− 0.081	0.317
	*95% confidence interval*	− 0.047–0.245	0.190–0.491	− 0.463–0.197	− 0.249–0.091	0.177–0.444
	*p-value*^*3*^	0.3	< 0.001	< 0.001	0.5	< 0.001
No clefting besides macrostomia, *n* = 148	*No. of patients*	147	106	143	113	146
	*Correlation coefficient*^*2*^	0.049	0.462	− 0.286	− 0.063	0.375
	*95% confidence interval*	− 0.114–0.209	0.298–0.600	− 0.430–0.128	− 0.245–0.123	0.226–0.506
	*p-value*^*3*^	0.7	< 0.001	0.002	0.7	< 0.001
No clefting at all, *n* = 122	*No. of patients*	122	88	117	92	120
	*Correlation coefficient*^*2*^	0.120	0.214	− 0.021	0.121	0.224
	*95% confidence interval*	− 0.059–0.292	0.004–0.405	− 0.212–0.161	− 0.086–0.318	0.047–0.388
	*p-value*^*3*^	0.4	0.14	> 0.9	0.5	0.063

^1^Pruzansky-Kaban scores

^2^Spearman Rank correlation coefficient

^3^*p*-value adjusted for multiple testing according to the Benjamini–Hochberg method

## Discussion

In this study, objective measurements derived from 3D photographs were used to assess the association between facial morphology and craniofacial anomalies to identify clinically relevant clusters of patients based on these characteristics.

Two clusters of patients were identified. A more severe facial phenotype, corresponding to more facial hypoplasia, was seen in patients with anomalies in at least two of the following groups: ocular anomalies, skin adnexa-related anomalies, clefting and aural atresia (cluster 2). Milder facial phenotypes, or less facial hypoplasia, were seen in individuals with anomalies in up to two of these groups (cluster 1). Previous studies have not found clear clusters based on patients classified using the OMENS scale, nor when assessing characteristics associated with Goldenhar’s syndrome, such as epibulbar dermoids [8, 9]. Weak correlations have been found between (1) CL/P and structures of the first pharyngeal arch, (2) skin tags and ocular anomalies, and (3) ocular anomalies and the Pruzansky-Kaban score [8]. In the current study, patients with more craniofacial anomalies (cluster 2) were similarly weakly correlated with higher Pruzansky-Kaban scores and higher soft tissue scores. However, in contrast to previous studies, the current study used comprehensive and objective measures for assessed facial hypoplasia, negating reliance on OMENS scores for clustering, and grouped associated craniofacial malformations instead of selecting individual malformations (e.g. epibulbar dermoids), which may explain the slight discrepancy with previous results.

The subgroup analyses on the effect of clefting on these results revealed several interesting points. In the macrostomia subgroup, where patients with CL/P were excluded, cluster assignment remained associated with facial morphology, other craniofacial anomalies, as well as the Pruzansky-Kaban score. These effects were most pronounced in the macrostomia subgroup analysis. These results seem to be in agreement with two other studies, in which macrostomia was significantly associated with the mandible subscale of the OMENS [26, 27], although nother larger study found no significant correlations between macrostomia and the Pruzansky-Kaban score [8]. The current results perhaps support a more nuanced conclusion: patients with macrostomia have more facial hypoplasia and higher Pruzansky-Kaban scores, especially if other craniofacial anomalies, such as ocular- and skin-adnexa related anomalies are also present. Another potentially surprising finding was a negative correlation between ear scores and cluster assignments in both the primary analysis and in the macrostomia subanalysis. This finding was unexpected as ear scores were not correlated with increasing severity of either ASI or FS scores in our previous analysis assessing morphological patterns in patients with CFM compared to controls [12]. Lobule type microtia is the most commonly seen form of microtia in both patients with CFM and isolated microtia [8, 28], supporting the results of our previous study that the type of microtia is not necessarily directly related to the severity of facial morphology. Instead, the current results suggest a separate cluster of patients with CFM and a mild malformation of the ear, who may have more associated craniofacial anomalies as well as a more severe facial phenotype, especially if clefting is present. In the subgroup excluding all types of clefting, statistically significant cluster differences were mainly related to the presence of craniofacial anomalies. However, cluster 2 in this analysis was small, possibly affecting the reliability of these results. Larger study populations would be needed to further assess and confirm these associations.

It is interesting to note that VPD was still observed in both subgroups of patients without intraoral clefting, although its incidence was not clearly associated with cluster assignment in the subgroups. A previous study found soft palate dysfunction and VPD in patients with CFM and isolated microtia without a history of cleft palate, as well as a weak correlation between mandible scores and VPD [7]. Another study also recorded significant correlations between the mandible score and VPD [29]. In the current study, higher ASI and FS scores were seen in the midface of patients with VPD compared to those without, although these differences were not statistically significant in the subgroups that excluded clefting after adjusting for multiple testing (Online Resource 9). Nevertheless, these results may suggest that even in the absence of clefting, patients with VPD may have morphological differences and increased asymmetry in the midface compared to patients without VPD. Considering the limitations involving the small subgroup size, larger future studies may focus on these morphological differences to clarify which patients require preferential screening.

Clinically, the findings of the current study could suggest that patients with more severely affected facial features, or more facial hypoplasia, should be screened for other associated craniofacial anomalies. Radiographic imaging for the classification of mandibular hypoplasia is often delayed [3] and the reliability of several OMENS subscales can be debated [10, 30]. 3D photography offers a promising tool for objectively assessing facial hypoplasia. In a previous study, significant correlations between a principal components model for CFM and the Pruzansky-Kaban score were found, and an increasing pattern of orbital involvement was seen, suggesting the applicability of 3D photography in assessing and classifying the severity of facial involvement in CFM [12, 31]. The current study further suggests that 3D photography, coupled with morphological analyses, could potentially be used to distinguish between patients unlikely to need further screening for associated craniofacial anomalies, and those more likely to need additional screening in the future. Future studies could expand on and reinforce these results by focusing on implementing artificial intelligence-based frameworks for objective and robust automated diagnostics and classification [32].

Recently, such frameworks have been implemented in syndrome diagnostics by identifying gene-linked syndromes based on facial shape [33, 34]. Several genes have been implicated in the etiology of CFM and microtia, but no definitive causality has been established [35]. Artificial intelligence-based frameworks have the potential to improve phenotype-genotype assessments [34], and could be interesting in exploring the phenotype-genotype association of 3D facial shape and candidate genes in CFM and microtia.

The retrospective nature of this study is its most important limitation, as it leads to a reliance on complete reporting in medical charts. Nerve weakness examinations, especially relating to the uvula, were not always clearly reported in medical charts. Additionally, detecting middle- and inner ear anomalies, which requires temporal bone CT imaging that is performed at a higher resolution compared to a facial CT, were not routinely performed in all patients. This may have influenced the results especially related to the identification of these groups of anomalies. Future (prospective) studies may improve on our results by investigating the additional effects of nerve weakness, as well as types of middle- and inner ear anomalies on facial morphology and clustering tendency. As a European-based study, the results may also not be directly generalizable globally. The clustering tendency of this dataset was weak (Online Resource 8), suggesting some overlap in cluster characteristics. For example, patients with two different craniofacial anomalies could be assigned to either cluster (Table 2). This could suggest other, more specific clustering that should be investigated in larger cohort studies. Furthermore, this study included fewer patients than previous studies due to the relative novelty of using 3D photography in routine clinical practice. As this study focused on the association of facial morphology and other craniofacial malformations, the study size was limited to patients with usable 3D photographs. Although medical stereophotogrammetry systems may not currently be accessible to all units due to the associated costs, smartphone-based systems offer potential future alternatives for accessible 3D photography [36].

## Conclusion

This study assessed associations between objectively measured 3D facial morphology and concurrent craniofacial malformations in patients with CFM and microtia. Two clusters were identified based on facial morphology and anomalies in the following groups: ocular anomalies, skin adnexa-related anomalies, clefting (including macrostomia) and aural atresia. Patients with anomalies in two or more of these groups had more severe facial phenotypes, corresponding to more facial hypoplasia, while patients with anomalies in two or fewer of these groups had milder facial phenotypes, corresponding to less facial hypoplasia. This may suggest that 3D photography could be a promising tool for identifying patients that should be screened for these concurrent craniofacial anomalies. Future studies could implement artificial intelligence-based frameworks to expand on these results.

## Supplementary information

Below is the link to the electronic supplementary material.

ESM 1 (DOCX 23.6 KB)

ESM 2 (DOCX 1.61 MB)

ESM 3 (DOCX 21.4 KB)

ESM 4 (DOCX 15.0 KB)

ESM 5 (DOCX 306 KB)

ESM 6 (DOCX 306 KB)

ESM 7 (DOCX 647 KB)

ESM 8 (DOCX 211 KB)

ESM 9 (DOCX 309 KB)

## Data Availability

Data may be made available on request.

## Abbreviations

CFM: Craniofacial microsomia
CL/P: Cleft lip and/or palate
VPD: Velopharyngeal dysfunction
OMENS: Orbit, mandible, ear, nerve and soft tissue classification tool
3D: Three-dimensional
PAT-CFM: Phenotypic assessment tool for craniofacial microsomia
STROBE: Strengthening the reporting of observational studies in epidemiology
ASI: Asymmetry index
FS: Facial signature
PCA: Principal components analysis
PC: Principal component
CT: Computed tomography
