# Supplementary material for: Clusters in craniofacial microsomia and microtia according to facial morphology and craniofacial anomalies
Source: Eur J Pediatr. 2026 Apr 24;185(5):298. doi: 10.1007/s00431-026-06973-9 (PMC13109105; doi:10.1007/s00431-026-06973-9)
Supplement: Supplementary file 2 — (DOCX 1.61 MB) [file 431_2026_6973_MOESM2_ESM.docx]

**Online Resource 2** A visual representation of loading scores for the principal components distinguishing patients with CFM from individuals without craniofacial conditions. Loading scores represent correlations between the original FS scores and principal component scores. Positive loadings (yellow) indicate a positive correlation with the original FS scores, and negative loadings (purple) represent a negative correlation. These components illustrate both relatively symmetrical (PC13, PC19, PC23) and relatively asymmetrical facial shape patterns involving mainly the orbits, mandible and soft tissues of the cheeks. These principal components were previously found to correlate to Orbit, Mandible, Ear, Nerve and Soft tissue (OMENS) scores^1^

*
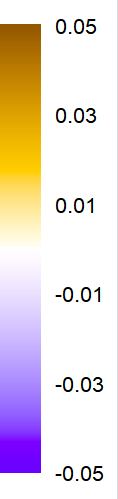
*

| **PC13** | **PC14** | **PC15** | **PC19** | **PC20** |
| --- | --- | --- | --- | --- |
| 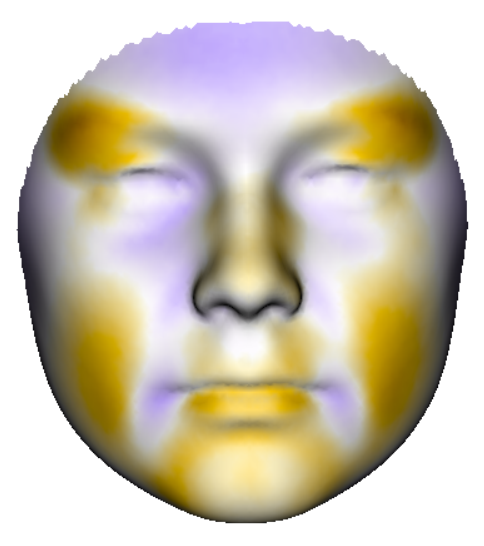 | 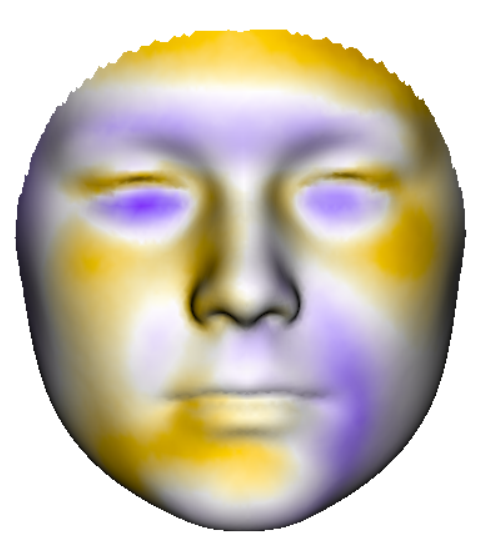 | 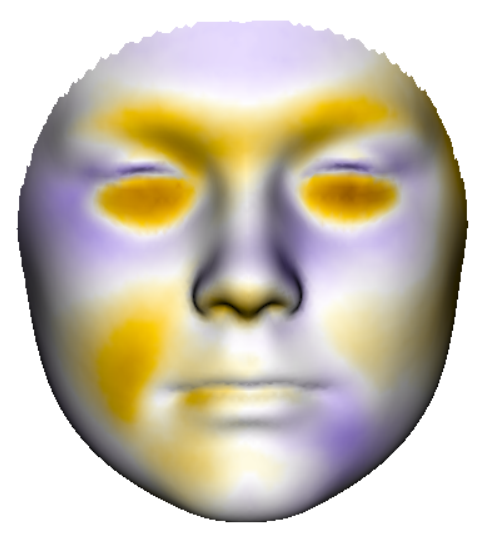 | 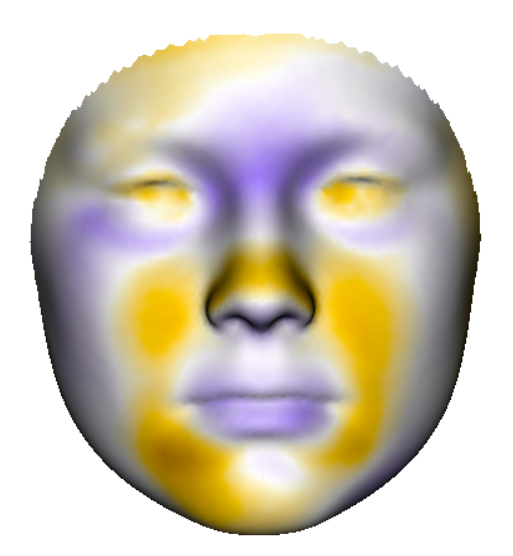 | 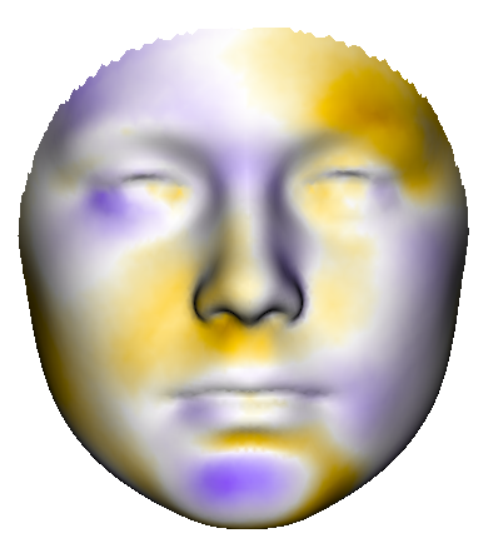 |

| **PC23** | **PC30** | **PC33** | **PC34** |
| --- | --- | --- | --- |
| 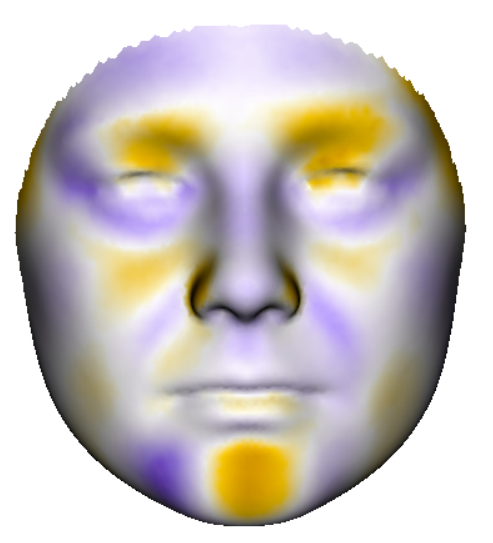 | 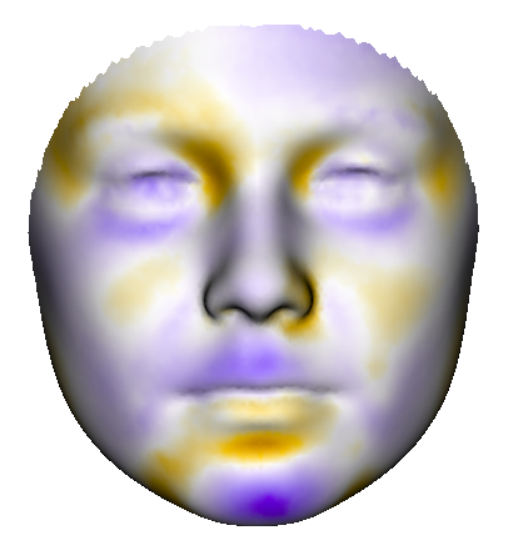 | 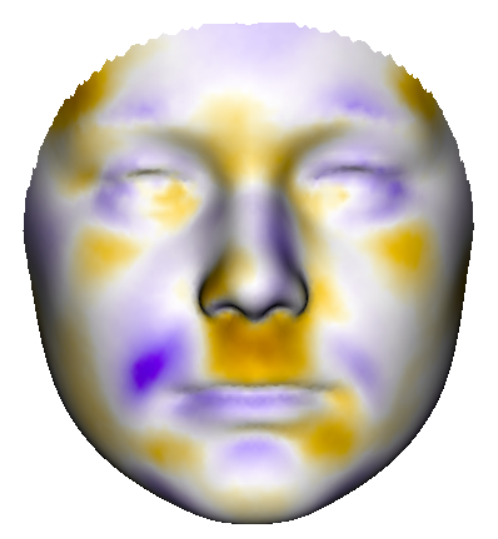 | 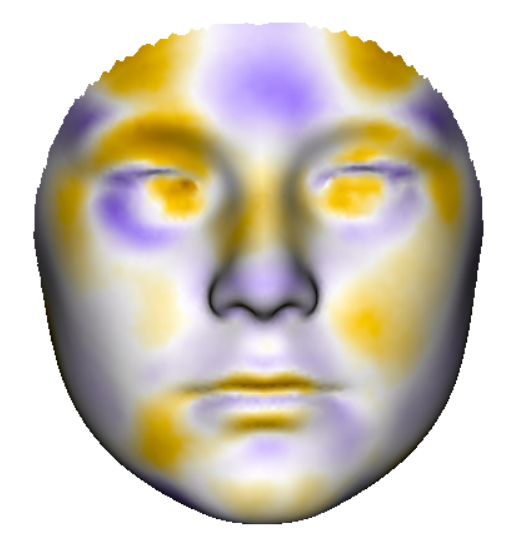 |

1. Ronde EM et al. Three-Dimensional Facial Morphology in Patients with Craniofacial Microsomia and Microtia. Plast Reconstr Surg. 2025 Aug 1;156(2):304-315. doi: 10.1097/PRS.0000000000011831.
