# Supplementary material for: Clusters in craniofacial microsomia and microtia according to facial morphology and craniofacial anomalies
Source: Eur J Pediatr. 2026 Apr 24;185(5):298. doi: 10.1007/s00431-026-06973-9 (PMC13109105; doi:10.1007/s00431-026-06973-9)
Supplement: Supplementary file 3 — (DOCX 21.4 KB) [file 431_2026_6973_MOESM3_ESM.docx]

**Online Resource 3** All craniofacial anomalies

|  | **Total no. patients (%)** | **Side of anomaly** | | |
| --- | --- | --- | --- | --- |
|  |  | *Unilateral (right)* | *Unilateral (left)* | *Bilateral* |
| **Ocular anomalies** | 44 (25) | 12 (7) | 17 (10) | 15 (8) |
| Colobomata | 8 (5) | 2 (1) | 3 (2) | 3 (2) |
| Upper lid | 4 (2) | 1 (1) | 2 (1) | 1 (1) |
| Lower lid | 2 (1) | 0 | 1 (1) | 1 (1) |
| Iris | 2 (1) | 1 (1) | 0 | 1 (1) |
| Chorioretinal | 2 (1) | 1 (1) | 1 (1) | 0 |
| Strabismus | 25 (14) | 9 (5) | 8 (5) | 8 (5) |
| Exotropia | 13 (7) | 5 (3) | 5 (3) | 3 (2) |
| Esotropia | 9 (5) | 4 (2) | 3 (2) | 2 (1) |
| Type unknown | 3 (2) | 0 | 0 | 3 (2) |
| Epibulbar dermoids | 12 (7) | 3 (2) | 6 (2) | 3 (2) |
| Micro- or anophthalmia | 4 (2) | 2 (1) | 2 (1) | 0 |
| Congenital cataracts | 2 (1) | 1 (1) | 0 | 1 (1) |
| Pterygium | 2 (1) | 0 | 2 (1) | 0 |
| Other^1^ | 6 (3) | 3 (2) | 3 (2) | 1 (1) |
| **Skin adnexa-related anomalies** | 101 (56) | 30 (17) | 37 (21) | 34 (19) |
| Skin tags | 92 (51) | 30 (17) | 38 (21) | 24 (13) |
| Preauricular tags | 82 (46) | 26 (15) | 34 (19) | 22 (12) |
| Ear tags | 15 (8) | 6 (3) | 7 (4) | 2 (1) |
| Facial tags | 16 (9) | 6 (3) | 8 (5) | 3 (2) |
| Pits or fistulas | 42 (24) | 15 (8) | 19 (11) | 8 (5) |
| Preauricular pits/fistulas | 24 (13) | 9 (5) | 12 (6) | 3 (2) |
| Ear pits/fistula | 14 (8) | 5 (3) | 8 (5) | 1 (1) |
| Facial pits/fistulas | 9 (5) | 2 (1) | 3 (2) | 4 (2) |
| Dermoid cysts | 3 (2) | 3 (2) | 0 | 0 |
| Mirror ear | 2 (1) | 2 (1) | 0 | 0 |
| Hemangiomas^2^ | 4 (2) | 1 (1) | 2 (1) | 0 |
| LV anomaly | 1 (1) | 1 (1) | 0 | 0 |
| **Nerve weakness** | 94 (48) | 40 (22) | 30 (17) | 24 (13) |
| Facial nerve weakness | 79 (44) | 40 (22) | 36 (20) | 3 (2) |
| Uvula deviation^2^ | 37 (21) | 17 (10) | 18 (10) | 0 |
| Tongue deviation | 4 (2) | 4 (2) | 0 | 0 |
| Other^3^ | 2 (1) | 0 | 1 (1) | 1 (1) |
| **Clefting** | 57 (32) | 16 (9) | 12 (7) | 29 (16)^4^ |
| Macrostomia | 35 (20) | 18 (10) | 16 (9) | 1 (1) |
| Cleft lip | 3 (2) | 2 (1) | 0 | 1 (1) |
| Cleft palate | 11 (6) | 2 (1) | 1 (1) | 8 (5)^4^ |
| Submucous cleft palate | 15 (8) | n/a | n/a | 15 (8)^4^ |
| Bifid uvula | 12 (7) | n/a | n/a | 12 (7) |
| Other^5^ | 2 (1) | 0 | 0 | 2 (1) |
| **Other intraoral anomalies** | 7 (4) | 2 (1) | 1 (1) | 4 (2) |
| Tongue hypoplasia | 2 (1) | 2 (1) | 0 | 0 |
| Other^6^ | 5 (3) | 0 | 1 (1) | 3 (2) |

*Continued on next page*

|  | **Total no. patients (%)** | **Side of anomaly** | | |
| --- | --- | --- | --- | --- |
|  |  | *Unilateral (right)* | *Unilateral (left)* | *Bilateral* |
| **Aural atresia** | 146 (82) | 76 (43) | 55 (31) | 15 (8) |
| Complete atresia | 86 (48) | 41 (23) | 39 (22) | 6 (3) |
| Partial atresia | 52 (29) | 30 (17) | 17 (10) | 5 (3) |
| Unknown degree | 12 (7) | 9 (5) | 3 (2) | 0 |
| **Middle ear anomalies** | 75 (42) | 27 (15) | 29 (16) | 19 (11) |
| Congenital cholesteatoma | 5 (3) | 4 (2) | 1 (1) | 0 |
| Smaller middle ear | 31 (17) | 15 (8) | 13 (7) | 3 (2) |
| Ossicular malformation | 65 (36) | 28 (16) | 23 (13) | 14 (8) |
| Dysplasia or aplasia of the stapes | 52 (29) | 22 (12) | 23 (13) | 7 (4) |
| Dysplasia or aplasia of the incus | 52 (29) | 22 (12) | 24 (13) | 6 (3) |
| Dysplasia or aplasia of the malleolus | 50 (28) | 23 (13) | 20 (11) | 7 (4) |
| Incudomalleolar joint fusion | 18 (10) | 9 (5) | 9 (5) | 0 |
| Incudostapedial joint fusion | 1 (1) | 0 | 1 (1) | 0 |
| Fusion malleolus with tympanic plate | 6 (3) | 3 (2) | 2 (1) | 1 (1) |
| Ossicular malformation, NS | 3 (2) | 1 (1) | 1 (1) | 1 (1) |
| Poor/no mastoid pneumatization | 36 (20) | 15 (8) | 19 (11) | 2 (1) |
| Atresia of the round window | 7 (4) | 1 (1) | 5 (3) | 1 (1) |
| Atresia of the oval window | 20 (11) | 9 (5) | 9 (5) | 2 (1) |
| Aberrant course of N. VII | 34 (19) | 16 (9) | 15 (8) | 3 (2) |
| Aberrant course of carotid artery | 6 (3) | 2 (1) | 4 (2) | 0 |
| High riding jugular bulb | 11 (6) | 4 (2) | 4 (2) | 3 (2) |
| Overhanging dura | 5 (3) | 1 (1) | 2 (2) | 0 |
| Soft tissue in middle ear | 2 (1) | 1 (1) | 1 (1) | 0 |
| Complete atresia | 5 (3) | 1 (1) | 4 (2) | 0 |
| Unspecified | 5 (3) | 2 (1) | 2 (1) | 1 (1) |
| Other^7^ | 3 (2) | 1 (1) | 2 (1) | 0 |
| **Inner ear anomalies** | 33 (18) | 8 (5) | 12 (7) | 13 (7) |
| Cochlear malformation | 13 (7) | 1 (1) | 7 (4) | 5 (3) |
| Narrow internal auditory canal | 10 (5) | 2 (1) | 8 (5) | 0 |
| Dysplasia of endolymphatic canal or sac | 2 (1) | 0 | 2 (1) | 0 |
| Dysplasia or hypoplasia of semicircular canals | 20 (11) | 4 (2) | 9 (5) | 7 (4) |
| Vestibular dysplasia or hypoplasia | 8 (4) | 1 (1) | 4 (2) | 3 (2) |
| N VIII hypoplasia | 5 (3) | 2 (1) | 3 (2) | 0 |
| Unspecified | 6 (3) | 3 (2) | 1 (1) | 2 (1) |
| Other^8^ | 2 (1) | 1 (1) | 1 (1) | 0 |
| ^1^ Entropion, membrane of Hasner, sclerocornea, heterochromiairidis, distiachasis, dacryostenosis, blepharophimosis | | | | |
| ^2^ Unknown side: hemangioma (1) and uvula deviation (2). | | | | |
| ^3^ Anosmia, NIV paresis | | | | |
| ^4^ Central clefting categorized under bilateral. Combinations including central clefting categorized as bilateral. | | | | |
| ^5^ Bifid nose tip, bifid tongue tip. | | | | |
| ^6^ Ranula, short lingual frenulum, hypertrophic upper frenulum, palatal teeth, one unknown. | | | | |
| ^7^ Malformed stapedial ligament, Eustachian tube connection to sphenoid sinus, dysplasia of the m. stapedius | | | | |
| ^8^  Other inner: Absence of NVII complex, common origin facial and trigeminal nerve | | | | |
