# Supplementary material for: Clusters in craniofacial microsomia and microtia according to facial morphology and craniofacial anomalies
Source: Eur J Pediatr. 2026 Apr 24;185(5):298. doi: 10.1007/s00431-026-06973-9 (PMC13109105; doi:10.1007/s00431-026-06973-9)
Supplement: Supplementary file 4 — (DOCX 15.0 KB) [file 431_2026_6973_MOESM4_ESM.docx]

**Online Resource 4.** Speech and language-, vision- and hearing difficulties.

|  | **Total N (%)** | **Unilateral (right)** | **Unilateral (left)** | **Bilateral** |
| --- | --- | --- | --- | --- |
| **Speech difficulties** | 87 (49) | n/a | n/a | n/a |
| VPD | 26 (15) | n/a | n/a | n/a |
| Speech sound disorders | 19 (11) | n/a | n/a | n/a |
| Motor speech disorders | 8 (5) | n/a | n/a | n/a |
| Expressive language difficulties | 6 (3) | n/a | n/a | n/a |
| Verbal dyspraxia | 2 (1) | n/a | n/a | n/a |
| Speech/language delay, NS | 35 (20) | n/a | n/a | n/a |
| Comprehension difficulties, NS | 6 (3) | n/a | n/a | n/a |
| Unspecified | 15 (8) | n/a | n/a | n/a |
| Other^1^ | 4 (2) | n/a | n/a | n/a |
| **Hearing loss** | 150 (84) | 67 (37) | 52 (29) | 31 (17) |
| Conductive | 125 (70) | 59 (33) | 47 (26) | 19 (11) |
| Sensorineural | 8 (4) | 4 (2) | 4 (2) | 0 |
| Mixed | 19 (11) | 8 (5) | 5 (3) | 6 (3) |
| Unspecified | 4 (2) | 2 (1) | 2 (1) | 0 |
| **Vision impairment** | 48 (27)^2^ | 6 (3) | 11 (6) | 30 (17) |
| Unspecified | 11 (6) | 1 (1) | 1 (1) | 9 (5) |
| Amblyopia | 15 (8)^2^ | 4 (2) | 7 (4) | 3 (2) |
| Anisometropia | 4 (2) | 0 | 2 (1) | 2 (1) |
| Hypermetropia | 12 (7) | 10 (6) | 1 (1) | 1 (1) |
| Myopia | 5 (3) | 1 (1) | 0 | 4 (2) |
| Astigmatism | 4 (2) | 1 (1) | 1 (1) | 2 (1) |
| Other^3^ | 3 (2) | 0 | 0 | 3 (2) |
| ^1^ No language development due to hearing, insufficient vocabulary, tracheostomy, frenulum | | | | |
| ^2^ Laterality unspecified in one patient. | | | | |
| ^3^ Unspecified refractive impairment, nystagmus, convergence insufficiency. | | | | |
