# Supplementary material for: Clusters in craniofacial microsomia and microtia according to facial morphology and craniofacial anomalies
Source: Eur J Pediatr. 2026 Apr 24;185(5):298. doi: 10.1007/s00431-026-06973-9 (PMC13109105; doi:10.1007/s00431-026-06973-9)
Supplement: Supplementary file 5 — (DOCX 306 KB) [file 431_2026_6973_MOESM5_ESM.docx]

**Online Resource 5** Asymmetry index (ASI) scores for unilateral patients with and without craniofacial anomalies and speech- and language, hearing and vision difficulties per region.

| **Regions^1^** |  | **1**  **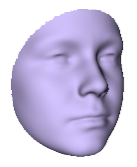** | **2**  **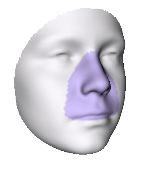** | **3**  **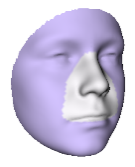** | **4**  **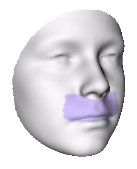** | **5**  **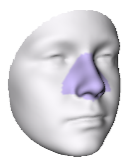** | **6**  **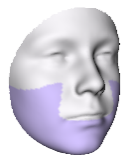** | **7**  **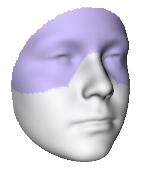** | **8**  **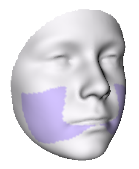** | **9**  **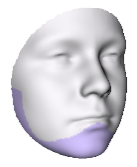** | **10**  **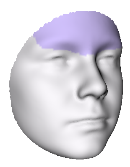** | **11**  **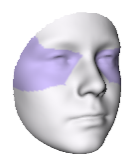** |
| --- | --- | --- | --- | --- | --- | --- | --- | --- | --- | --- | --- | --- |
| *Ocular anomalies* | **Yes,** n=41 | 1.78◦ | 1.17*** | 1.82◦ | 1.20*** | 1.05** | 1.81 | 1.32** | 1.45 | 1.72 | 1.28* | 1.39** |
|  |  | (1.21-2.13) | (0.86-1.60) | (1.17-2.13) | (0.82-1.84) | (0.67-1.62) | (1.08-2.54) | (1.07-1.70) | (1.10-2.32) | (1.13-2.76) | (0.75-1.66) | (1.05-1.75) |
|  | **No,** n=119 | 1.30 | 0.88 | 1.36 | 0.87 | 0.74 | 1.53 | 1.13 | 1.37 | 1.72 | 1.03 | 1.13 |
|  |  | (0.96-1.97) | (0.64-1.17) | 0.97-2.12) | (0.58-1.30) | (0.56-1.02) | (0.90-2.49) | (0.77-1.45) | (0.77-2.01) | (1.00-2.77) | (0.65-1.41) | (0.77-1.46) |
| *Skin adnexa-related anomalies* | **Yes,** n=85 | 1.62 | 0.92 | 1.67 | 0.92 | 0.77 | 1.64◦ | 1.15 | 1.58* | 1.84 | 1.06 | 1.20 |
|  |  | (1.02-2.14) | (0.65-1.27) | (1.08-2.27) | (0.66-1.51) | (0.60-1.10) | (1.10-2.73) | (0.80-1.48) | (0.93-2.32) | (1.11-3.07) | (0.64-1.51) | (0.79-1.51) |
|  | **No,** n=75 | 1.29 | 0.95 | 1.36 | 0.91 | 0.80 | 1.53 | 1.24 | 1.23 | 1.69 | 1.07 | 1.21 |
|  |  | (0.99-1.90) | (0.66-1.26) | (1.01-2.04) | (0.65-1.46) | (0.54-1.18) | (0.88-2.34) | (0.88-1.53) | (0.75-1.75) | (0.97-2.34) | (0.74-1.46) | (0.90-1.57) |
| *Nerve weakness* | **Yes,** n=84 | 1.39 | 0.93 | 1.47 | 0.94 | 0.78 | 1.52 | 1.27◦ | 1.41 | 1.71 | 1.09 | 1.25◦ |
|  |  | (1.07-2.10) | (0.65-1.28) | (1.09-2.12) | (0.66-1.57) | (0.56-1.30) | (1.10-2.53) | (0.95-1.57) | (0.90-2.00) | (1.12-2.73) | (0.75-1.52) | (0.90-1.63) |
|  | **No**, n=76 | 1.36 | 0.92 | 1.45 | 0.86 | 0.83 | 1.62 | 1.10 | 1.26 | 0.79 | 1.00 | 1.06 |
|  |  | (0.93-2.06) | (0.68-1.26) | (0.92-2.17) | (0.65-1.42) | (0.58-1.11) | (0.88-2.48) | (0.78-1.45) | (0.78-2.30) | (0.91-2.82) | (0.59-1.41) | (0.78-1.44) |
| *Clefting* | **Yes,** n=48 | 2.06*** | 1.20*** | 2.12*** | 1.49*** | 0.89* | 2.49*** | 1.40** | 2.25*** | 2.65*** | 1.31** | 1.40** |
|  |  | (1.57-2.50) | (0.91-1.61) | (1.55-2.59) | (0.91-1.77) | (0.66-1.34) | (1.51-3.31) | (1.06-1.69) | (1.34-2.66) | (1.54-3.66) | (0.88-1.71) | (1.13-1.64) |
|  | **No,** n=112 | 1.26 | 0.83 | 1.31 | 0.80 | 0.74 | 1.38 | 1.10 | 1.18 | 1.52 | 0.97 | 1.08 |
|  |  | (0.94-1.70 | (0.61-1.13) | (0.94-1.82) | (0.57-1.15) | (0.54-1.06) | (1.86-2.20) | (0.79-1.43) | (0.76-1.70) | (0.91-2.23) | (0.63-1.38) | (0.78-1.44) |
| *Aural atresia* | **Yes**, n=129 | 1.36 | 0.95 | 1.45 | 0.93 | 0.8 | 1.53 | 1.18 | 1.4 | 1.71 | 1.07 | 1.2 |
|  |  | (1.01-2.09) | (0.65-1.32) | (1.03-2.15) | (0.63-1.52) | (0.58-1.21) | (0.97-2.54) | (0.85-1.52) | (0.84-2.16) | (1.01-2.76) | (0.70-1.50) | (0.80-1.54) |
|  | **No**, n=31 | 1.63 | 0.87 | 1.62 | 0.87 | 0.75 | 1.81 | 1.22 | 1.22 | 1.88 | 0.97 | 1.21 |
|  |  | (1.01-1.91) | (0.75-1.17) | (1.07-2.05) | (0.69-1.41) | (0.54-0.97) | (1.07-2.44) | (0.94-1.47) | (0.86-2.01) | (1.17-2.92) | (0.58-1.31) | (0.97-1.56) |
| *Middle ear anomalies* | **Yes**, n=65 | 1.37 | 0.93 | 1.44 | 0.88 | 0.78 | 1.44 | 1.27 | 1.32 | 1.59 | 1.08 | 1.24 |
|  |  | (1.03-2.01) | (0.66-1.39) | (1.06-2.13) | (0.64-1.51) | (0.61-1.28) | (1.10-2.44) | (0.90-1.55) | (0.84-2.03) | (1.05-2.59) | (0.76-1.58) | (0.82-1.57) |
|  | **No,** n=95 | 1.5 | 0.94 | 1.62 | 0.94 | 0.8 | 1.73 | 1.14 | 1.45 | 1.86 | 1.04 | 1.2 |
|  |  | (0.99-2.12) | (0.66-1.24) | (1.01-2.13) | (0.66-1.50) | (0.54-1.06) | (0.98-2.54) | (0.84-1.46) | (0.86-2.25) | (1.04-2.87) | (0.64-1.43) | (0.84-1.51) |

*Continued on next page*

| **Regions^1^** |  | **1**  **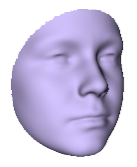** | **2**  **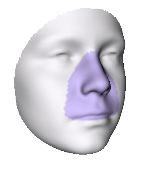** | **3**  **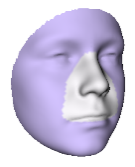** | **4**  **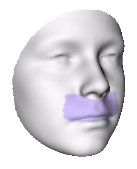** | **5**  **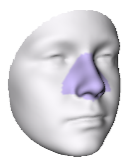** | **6**  **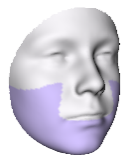** | **7**  **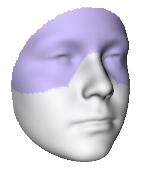** | **8**  **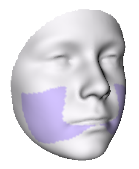** | **9**  **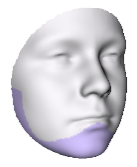** | **10**  **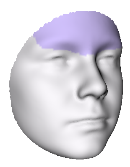** | **11**  **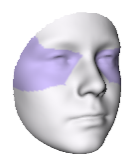** |  |
| --- | --- | --- | --- | --- | --- | --- | --- | --- | --- | --- | --- | --- | --- |
| *Inner ear anomalies* | **Yes,** n=28 | 1.58 | 1.18* | 1.6 | 1.05 | 1.10** | 1.54 | 1.33 | 1.48 | 1.64 | 1.05 | 1.39* |  |
|  |  | (1.16-2.21) | (0.80-2.01) | (1.16-2.27) | (0.78-1.86) | (0.72-1.99) | (1.17-2.65) | (1.02-1.70) | (1.08-2.35) | (1.15-2.70) | (0.74-1.66) | (1.13-1.71) |  |
|  | **No**, n=132 | 1.36 | 0.93 | 1.44 | 0.88 | 0.76 | 1.57 | 1.15 | 1.38 | 1.74 | 1.06 | 1.18 |  |
|  |  | (0.97-1.94) | (0.65-1.21) | (1.00-2.09) | (0.63-1.48) | (0.55-1.05) | (0.98-2.48) | (0.81-1.47) | (0.82-2.00) | (1.02-2.77) | (0.68-1.44) | (0.78-1.49) |  |
| *Speech and language difficulties* | **Yes,** n=74 | 1.58* | 0.98◦ | 1.58◦ | 1.06* | 0.77 | 1.82◦ | 1.27 | 1.61* | 1.91◦ | 1.12* | 1.23 |  |
|  |  | (1.21-2.05) | (0.67-1.43) | (1.19-2.12) | (0.71-1.71) | (0.61-1.26) | (1.30-2.56) | (0.95-1.54) | (1.00-2.22) | (1.26-2.92) | (0.84-1.60) | (0.91-1.54) |  |
|  | **No,** n=86 | 1.23 | 0.89 | 1.32 | 0.82 | 0.83 | 1.38 | 1.14 | 1.19 | 1.52 | 0.97 | 1.19 |  |
|  |  | (0.89-2.08) | (0.64-1.20) | (0.93-2.14) | (0.59-1.26) | (0.55-1.05) | (0.85-2.47) | (0.74-1.46) | (0.75-2.01) | (0.89-2.70) | (0.56-1.37) | (0.78-1.56) |  |
| *Hearing difficulties* | **Yes,** n=132 | 1.37 | 0.95 | 1.46 | 0.94 | 0.80 | 1.54 | 1.17 | 1.41 | 1.70 | 1.07 | 1.20 |  |
|  |  | (1.02-2.13) | (0.65-1.33) | (1.06-2.16) | (0.63-1.52) | (0.57-1.27) | (1.05-2.54) | (0.86-1.55) | (0.88-2.24) | (1.04-2.76) | (0.70-1.51) | (0.82-1.57) |  |
|  | **No,** n=28 | 1.52 | 0.87 | 1.63 | 0.84 | 0.74 | 1.80 | 1.23 | 1.17 | 1.98 | 1.01 | 1.24 |  |
|  |  | (0.93-1.88) | (0.76-1.10) | (0.96-2.02) | (0.69-1.15) | (0.59-0.94) | (0.94-2.42) | (0.86-1.34) | (0.81-1.97) | (1.05-2.86) | (0.59-1.32) | (0.91-1.47) |  |
| *Vision difficulties* | **Yes,** n=43 | 1.63 | 1.15** | 1.64 | 1.06* | 1.00** | 1.71 | 1.20 | 1.64 | 1.79 | 1.05 | 1.29 |  |
|  |  | (1.17-2.11) | (0.87-1.56) | (1.17-2.12) | (0.83-1.74) | (0.67-1.67) | (1.31-2.44) | (0.94-1.77) | (0.95-2.10) | (1.16-2.72) | (0.69-1.54) | (0.90-1.72) |  |
|  | **No,** n=117 | 1.33 | 0.87 | 1.44 | 0.85 | 0.75 | 1.52 | 1.15 | 1.37 | 1.67 | 1.07 | 1.19 |  |
|  |  | (0.97-2.06) | (0.64-1.20) | (0.98-2.15) | (0.61-1.42) | (0.56-1.04) | (0.92-2.55) | (0.81-1.48) | (0.83-2.16) | (1.01-2.87) | (0.70-1.49) | (0.78-1.50) |  |
| ^1^Median (interquartile range) | | | | | | | | | | | | | |
| ***p<0.001, **p<0.01, *p<0.05, ◦ p≤0.1, Mann Whitney U test. | | | | | | | | | | | | | |

*Continued from previous page*
