# Supplementary material for: Clusters in craniofacial microsomia and microtia according to facial morphology and craniofacial anomalies
Source: Eur J Pediatr. 2026 Apr 24;185(5):298. doi: 10.1007/s00431-026-06973-9 (PMC13109105; doi:10.1007/s00431-026-06973-9)
Supplement: Supplementary file 6 — (DOCX 306 KB) [file 431_2026_6973_MOESM6_ESM.docx]

**Online Resource 6**. Facial signature (FS) scores for patients with and without craniofacial anomalies and speech- and language, hearing and vision difficulties per region.

| **Regions^1^** |  | **1**  **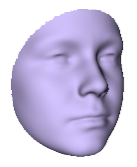** | **2**  **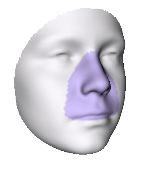** | **3**  **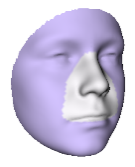** | **4**  **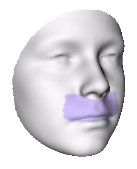** | **5**  **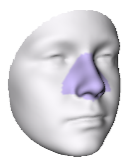** | **6**  **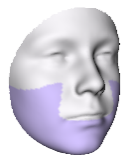** | **7**  **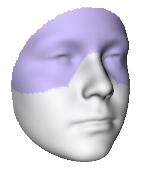** | **8**  **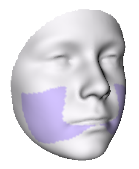** | **9**  **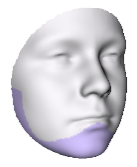** | **10**  **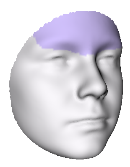** | **11**  **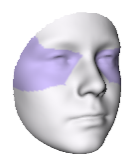** |
| --- | --- | --- | --- | --- | --- | --- | --- | --- | --- | --- | --- | --- |
| *Ocular anomalies* | **Yes,** n=44 | 1.55* | 1.37** | 1.61* | 1.30◦ | 1.33*** | 1.72* | 1.36 | 1.44** | 1.63 | 1.21 | 1.38* |
|  |  | (1.23-2.05) | (1.05-1.81) | (1.20-1.96) | (1.01-1.64) | (1.05-1.82) | (1.16-2.11) | (1.10-1.62) | (0.98-1.91) | (1.17-2.43) | (0.83-1.69) | (1.08-1.59) |
|  | **No,** n=135 | 1.34 | 1.11 | 1.37 | 1.08 | 1.30 | 1.37 | 1.24 | 1.14 | 1.43 | 1.14 | 1.18 |
|  |  | (1.09-1.57) | (0.90-1.40) | (1.09-1.64) | (0.86-1.50) | (0.85-1.36) | (1.08-1.81) | (0.95-1.54) | (0.89-1.54) | (1.12-1.93) | (0.83-1.60) | (0.94-1.44) |
| *Skin adnexa-related anomalies* | **Yes,** n=101 | 1.48** | 1.14 | 1.53*** | 1.14 | 1.11 | 1.70*** | 1.31◦ | 1.34** | 1.74** | 1.27** | 1.26 |
|  |  | (1.23-1.74) | (0.88-1.58) | (1.26-1.84) | (0.86-1.58) | (0.86-1.54) | (1.17-2.02) | (1.09-1.62) | (0.96-1.85) | (1.20-2.25) | (0.93-1.75) | (1.01-1.50) |
|  | **No,** n=78 | 1.29 | 1.21 | 1.28 | 1.22 | 1.15 | 1.22 | 1.25 | 1.07 | 1.30 | 1.03 | 1.16 |
|  |  | (1.05-1.51) | (1.02-1.45) | (1.03-1.51) | (0.93-1.51) | (0.93-1.40) | (1.01-1.54) | (0.91-1.49) | (0.91-1.34) | (1.09-1.66) | (0.78-1.33) | (0.93-1.46) |
| *Nerve weakness* | **Yes,** n=85 | 1.43 | 1.22◦ | 1.45 | 1.24 | 1.15 | 1.45 | 1.29 | 1.24 | 1.47 | 1.13 | 1.22 |
|  |  | (1.17-1.66) | (0.96-1.55) | (1.19-1.72) | (0.90-1.64) | (0.88-1.49) | (1.10-1.86) | (0.99-1.57) | (0.96-1.69) | (1.17-2.06) | (0.83-1.57) | (0.99-1.55) |
|  | **No**, n=94 | 1.35 | 1.08 | 1.37 | 1.09 | 1.04 | 1.37 | 1.29 | 1.14 | 1.51 | 1.21 | 1.22 |
|  |  | (1.10-1.67) | (0.98-1.42) | (1.06-1.73) | (0.86-1.49) | (0.86-1.41) | (1.06-1.92) | (0.96-1.60) | (0.87-1.57) | (1.11-2.10) | (0.83-1.78) | (0.93-1.43) |
| *Clefting* | **Yes,** n=48 | 1.59*** | 1.31* | 1.64*** | 1.25 | 1.22* | 1.81*** | 1.35* | 1.48*** | 1.92*** | 1.27* | 1.27* |
|  |  | (1.29-2.02) | (1.01-1.79) | (1.38-1.99) | (0.88-1.74) | (0.88-1.78) | (1.50-2.10) | (1.12-1.65) | (1.11-1.87) | (1.52-2.42) | (0.99-1.81) | (1.01-1.59) |
|  | **No,** n=112 | 1.30 | 1.12 | 1.31 | 1.08 | 1.07 | 1.26 | 1.23 | 1.08 | 1.33 | 1.08 | 1.18 |
|  |  | (1.08-1.51) | (0.92-1.39) | (1.04-1.56) | (0.87-1.49) | (0.95-1.36) | (1.04-1.72) | (0.92-1.54) | (0.88-1.45) | (1.06-1.79) | (0.82-1.50) | (0.92-1.45) |
| *Aural atresia* | **Yes,** n=146 | 1.36 | 1.19 | 1.41 | 1.16 | 1.14 | 1.39 | 1.31 | 1.19 | 1.45 | 1.18 | 1.26 |
|  |  | (1.14-1.67) | (0.94-1.48) | (1.14-1.73) | (0.88-1.58) | (0.88-1.50) | (1.10-1.84) | (1.00-1.59) | (0.93-1.66) | (1.16-2.05) | (0.84-1.67) | (0.98-1.49) |
|  | No, n=33 | 1.42 | 1.14 | 1.47 | 1.23 | 1.02 | 1.56 | 1.11 | 1.27 | 1.65 | 1.02 | 1.09 |
|  |  | (1.09-1.62) | (0.88-1.43) | (1.06-1.70) | (0.86-1.51) | (0.81-1.32) | (1.09-1.95) | (0.91-1.51) | (0.91-1.54) | (1.12-2.29) | (0.80-1.33) | (0.96-1.38) |
| *Middle ear anomalies* | **Yes**, n=75 | 1.35 | 1.2 | 1.41 | 1.16 | 1.15 | 1.43 | 1.24 | 1.19 | 1.44 | 1.12 | 1.28 |
|  |  | (1.12-1.78) | (0.94-1.70) | (1.13-1.86) | (0.86-1.70) | (0.91-1.56) | (1.03-1.94) | (1.00-1.64) | (0.93-1.86) | (1.08-2.09) | (0.84-1.54) | (1.00-1.57) |
|  | **No,** n=104 | 1.39 | 1.17 | 1.43 | 1.16 | 1.04 | 1.42 | 1.31 | 1.21 | 1.5 | 1.19 | 1.19 |
|  |  | (1.17-1.61) | (0.89-1.42) | (1.19-1.67) | (0.87-1.49) | (0.84-1.41) | (1.12-1.86) | (0.95-1.54) | (0.94-1.53) | (1.19-2.04) | (0.82-1.66) | (0.92-1.44) |

*Continued on next page*

*Continued from previous page*

| **Regions^1^** |  | **1**  **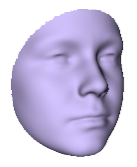** | **2**  **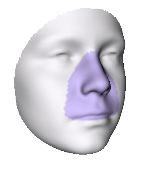** | **3**  **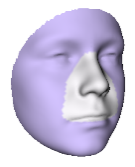** | **4**  **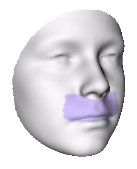** | **5**  **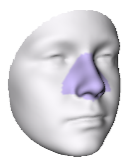** | **6**  **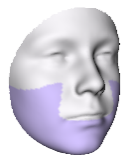** | **7**  **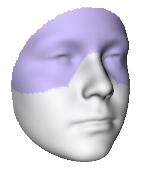** | **8**  **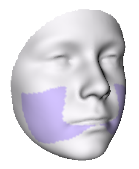** | **9**  **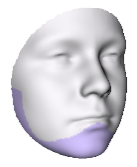** | **10**  **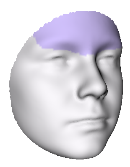** | **11**  **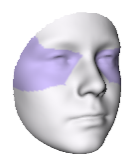** |  |
| --- | --- | --- | --- | --- | --- | --- | --- | --- | --- | --- | --- | --- | --- |
| *Inner ear anomalies* | **Yes**, n=33 | 1.47 | 1.36 | 1.53 | 1.29 | 1.34* | 1.55 | 1.43* | 1.44* | 1.57 | 1.28 | 1.31* |  |
|  |  | (1.24-2.04) | (1.01-1.71) | (1.25-1.87) | (0.87-1.70) | (0.86-1.69) | (1.12-2.03) | (1.17-1.66) | (1.11-1.90) | (1.09-2.05) | (0.97-1.81) | (1.18-1.73) |  |
|  | **No**, n=146 | 1.34 | 1.16 | 1.39 | 1.16 | 1.08 | 1.39 | 1.28 | 1.13 | 1.46 | 1.14 | 1.18 |  |
|  |  | (1.10-1.66) | (0.92-1.44) | (1.09-1.72) | (0.87-1.53) | (0.86-1.39) | (1.09-1.87) | (0.94-1.54) | (0.91-1.57) | (1.16-2.10) | (0.82-1.58) | (0.92-1.47) |  |
| *Speech/language difficulties* | **Yes,** n=87 | 1.47◦ | 1.26** | 1.48 | 1.23◦ | 1.16** | 1.50 | 1.32 | 1.22 | 1.57 | 1.20 | 1.24 |  |
|  |  | (1.14-1.73) | (1.02-1.66) | (1.16-1.77) | (0.89-1.64) | (0.95-1.61) | (1.09-1.94) | (1.04-1.60) | (0.94-1.69) | (1.17-2.12) | (0.94-1.65) | (0.99-1.55) |  |
|  | **No,** n=92 | 1.31 | 1.08 | 1.36 | 1.05 | 1.01 | 1.39 | 1.21 | 1.17 | 1.43 | 1.08 | 1.20 |  |
|  |  | (1.11-1.60) | (0.89-1.40) | (1.13-1.71) | (0.84-1.49) | (0.81-1.32) | (1.09-1.84) | (0.96-1.54) | (0.93-1.54) | (1.09-1.93) | (0.82-1.59) | (0.92-1.43) |  |
| *Hearing difficulties* | **Yes,** n=150 | 1.37 | 1.20 | 1.42 | 1.18 | 1.14 | 1.42 | 1.30 | 1.22 | 1.46 | 1.17 | 1.25 |  |
|  |  | (1.11-1.67) | (0.95-1.48) | (1.14-1.73) | (0.88-1.58) | (0.86-1.49) | (1.10-1.84) | (0.99-1.58) | (0.94-1.66) | (1.15-2.05) | (0.84-1.64) | (0.98-1.49) |  |
|  | **No,** n=29 | 1.37 | 1.07 | 1.41 | 1.02 | 1.00 | 1.53 | 1.11 | 1.05 | 1.65 | 1.02 | 1.09 |  |
|  |  | (1.19-1.62) | (0.87-1.43) | (1.23-1.70) | (0.80-1.49) | (0.84-1.32) | (1.09-1.95) | (0.96-1.54) | (0.87-1.34) | (1.13-2.29) | (0.82-1.33) | (0.96-1.35) |  |
| *Vision difficulties* | **Yes,** n=48 | 1.43 | 1.26 | 1.47 | 1.19 | 1.25* | 1.43 | 1.36* | 1.24 | 1.43 | 1.28* | 1.30 |  |
|  |  | (1.21-1.79) | (0.95-1.61) | (1.21-1.79) | (0.92-1.54) | (0.92-1.59) | (1.13-2.01) | (1.11-1.64) | (0.95-1.66) | (1.08-2.09) | (0.98-1.85) | (1.00-1.55) |  |
|  | **No,** n=131 | 1.35 | 1.13 | 1.39 | 1.16 | 1.04 | 1.43 | 1.21 | 1.19 | 1.50 | 1.11 | 1.18 |  |
|  |  | (1.09-1.66) | (0.91-1.46) | (1.08-1.72) | (0.86-1.55) | (0.86-1.41) | (1.07-1.86) | (0.95-1.53) | (0.92-1.62) | (1.15-2.08) | (0.81-1.54) | (0.95-1.45) |  |
| ^1^Median (interquartile range) | | | | | | | | | | | | | |
| ***p<0.001, **p<0.01, *p<0.05, ◦ p≤0.1, Mann Whitney U test. | | | | | | | | | | | | | |
