# Supplementary material for: Clusters in craniofacial microsomia and microtia according to facial morphology and craniofacial anomalies
Source: Eur J Pediatr. 2026 Apr 24;185(5):298. doi: 10.1007/s00431-026-06973-9 (PMC13109105; doi:10.1007/s00431-026-06973-9)
Supplement: Supplementary file 7 — (DOCX 647 KB) [file 431_2026_6973_MOESM7_ESM.docx]

**Online Resource 7** Principal component (PC) scores for patients with and without craniofacial anomalies and speech- and language, hearing and vision difficulties, per PCs from the logistic regression PCA model.

|  |  | **PC13** | **PC14** | **PC15** | **PC19** | **PC20** | **PC23** | **PC30** | **PC33** | **PC34** |
| --- | --- | --- | --- | --- | --- | --- | --- | --- | --- | --- |
| **Regions^1^** |  | **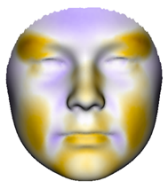** | **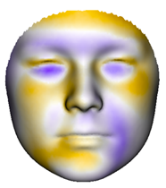** | **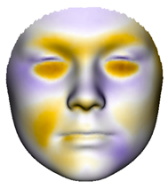** | **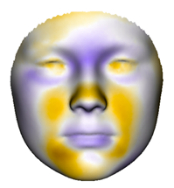** | **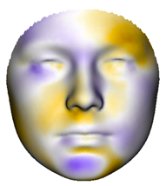** | **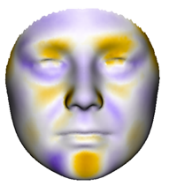** | **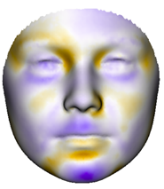** | **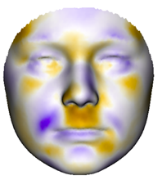** | **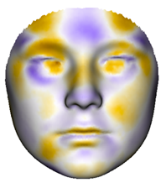** |
| *Ocular anomalies* | **Yes,** n=44 | -4.53 (12.95) | -6.30 (21.31) | 6.65 (20.24) | -1.58 (15.77) | -22.74 (22.91) ◦ | -9.85 (13.68) | 13.24 (12.81) | 3.37 (7.30) | -2.65 (6.22) |
|  | **No,** n=135 | -5.13 (11.83) | -9.08 (16.61) | 8.08 (16.78) | -2.53 (11.88) | -16.44 (17.01) | -7.24 (8.78) | 9.88 (11.21) | 1.67 (6.23) | -1.15 (5.87) |
| *Skin adnexa-related anomalies* | **Yes,** n=101 | -6.11 (12.34) | -8.65 (18.22) | 10.69 (17.44)* | -3.43 (13.63) | -22.06 (20.51)*** | -9.57 (10.44)* | 12.50 (12.04)* | 1.81 (6.54) | -1.87 (6.13) |
|  | **No,** n=78 | -3.52 (11.64) | -8.06 (17.49) | 3.89 (17.27) | -0.83 (11.83) | -12.71 (14.75) | -5.70 (9.56) | 8.39 (10.82) | 2.44 (6.54) | -1.07 (5.78) |
| *Nerve weakness* | **Yes,** n=94 | -5.51 (12.12) | -8.76 (16.88) | 5.89 (16.80) | -2.39 (10.88) | -16.15 (17.22) | -7.74 (9.30) | 9.24 (11.03) ◦ | 1.80 (6.76) | -1.44 (6.36) |
|  | **No**, n=85 | -4.39 (12.08) | -7.99 (18.97) | 9.75 (18.42) | -2.19 (14.89) | -20.02 (20.24) | -8.04 (11.21) | 12.33 (12.21) | 2.40 (6.29) | -1.61 (5.56) |
| *Clefting* | **Yes,** n=57 | -6.16 (11.23) | -10.09 (20.91) | 12.56 (21.87)* | -3.24 (16.78) | -27.24 (23.20)*** | -10.58 (11.02)* | 15.74 (12.91)*** | 2.32 (7.80) | -2.71 (6.34) ◦ |
|  | **No,** n=122 | -4.43 (12.46) | -7.60 (16.27) | 5.47 (14.85) | -1.86 (10.67) | -13.66 (14.48) | -6.62 (9.61) | 8.36 (10.29) | 1.98 (5.87) | -0.97 (5.74) |
| *Aural atresia* | **Yes**, n=146 | -4.54 (12.24) | -9.01 (17.89) | 6.91 (17.75) | -1.95 (12.76) | -18.60 (18.66) | -8.23 (10.36) | 10.48 (11.79) | 2.20 (6.62) | -1.53 (6.11) |
|  | **No**, n=33 | -6.91 (11.32) | -5.68 (17.74) | 11.33 (16.94) | -3.84 (13.60) | -15.28 (19.27) | -6.35 (9.57) | 11.71 (11.28) | 1.57 (6.18) | -1.46 (5.44) |
| *Middle ear anomalies* | **Yes**, n=75 | -5.64 (12.20) | -7.74 (19.31) | 7.51 (17.72) | -2.60 (14.72) | -16.49 (20.56) | -7.98 (10.72) | 9.48 (13.42) | 1.84 (6.79) | -2.14 (6.31) |
|  | **No,** n=104 | -4.51 (12.02) | -8.86 (16.81) | 7.88 (17.67) | -2.08 (11.49) | -19.07 (17.37) | -7.81 (9.90) | 11.60 (10.21) | 2.27 (6.36) | -1.07 (5.71) |
| *Inner ear anomalies* | **Yes,** n=33 | -3.01 (11.58) | -6.91 (23.56) | 8.70 (22.11) | -4.97 (14.83) | -16.40 (22.36) | -9.89 (12.73) | 11.34 (14.05) | 3.03 (6.76) | -2.77 (6.31) |
|  | **No**, n=146 | -5.43 (12.18) | -8.73 (16.38) | 7.50 (16.55) | -1.69 (12.40) | -18.35 (17.92) | -7.43 (9.56) | 10.57 (11.12) | 1.87 (6.48) | -1.24 (5.88) |

*Continued on next page*

*Continued from the previous page*

|  |  | **PC13** | **PC14** | **PC15** | **PC19** | **PC20** | **PC23** | **PC30** | **PC33** | **PC34** |
| --- | --- | --- | --- | --- | --- | --- | --- | --- | --- | --- |
| **Regions^1^** |  | **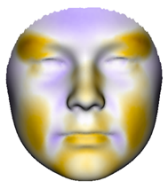** | **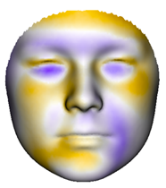** | **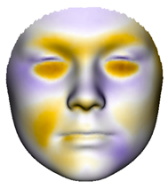** | **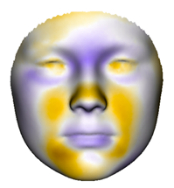** | **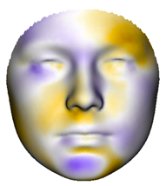** | **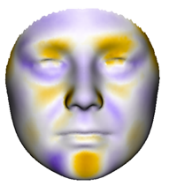** | **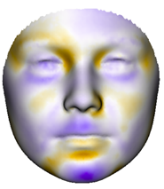** | **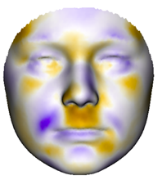** | **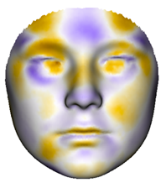** |
| *Speech and language difficulties* | **Yes,** n=87 | -6.13 (12.25) | -11.03 (19.59) ◦ | 7.58 (19.30) | -3.57 (14.65) | -19.43 (19.92) | -8.36 (10.75) | 11.82 (12.66) | 1.50 (7.08) | -1.57 (6.63) |
|  | **No,** n=92 | -3.90 (11.87) | -5.90 (15.76) | 7.86 (16.03) | -1.09 (10.95) | -16.62 (17.60) | -7.44 (9.74) | 9.66 (10.63) | 2.64 (5.95) | -1.47 (5.32) |
| *Hearing difficulties* | **Yes,** n=150 | -4.54 (12.01) | -9.50 (17.95) | 6.88 (17.49) | -2.44 (12.94) | -18.27 (19.48) | -8.03 (10.39) | 10.49 (11.89) | 2.40 (6.62) | -1.69 (6.17) |
|  | **No,** n=29 | -7.27 (12.39) | -2.66 (16.50) | 12.08 (18.12) | -1.54 (12.92) | -16.54 (14.70) | -7.11 (9.40) | 11.82 (10.61) | 0.49 (5.90) | -0.64 (4.83) |
| *Vision difficulties* | **Yes,** n=48 | -6.18 (11.90) | -5.93 (20.26) | 7.75 (18.88) | -1.73 (15.16) | -16.62 (22.67) | -9.34 (13.98) | 11.09 (13.58) | 2.43 (7.06) | -1.79 (6.73) |
|  | **No,** n=131 | -4.54 (12.16) | -9.30 (16.89) | 7.72 (17.24) | -2.51 (12.03) | -18.49 (17.18) | -7.35 (8.44) | 10.57 (10.95) | 1.96 (6.34) | -1.42 (5.70) |
| ^1^Mean (SD) | | | | | | | | | | |
| ***p<0.001, **p<0.01, *p<0.05, ◦ p≤0.1, two sample t-test | | | | | | | | | | |
