# Supplementary material for: Clusters in craniofacial microsomia and microtia according to facial morphology and craniofacial anomalies
Source: Eur J Pediatr. 2026 Apr 24;185(5):298. doi: 10.1007/s00431-026-06973-9 (PMC13109105; doi:10.1007/s00431-026-06973-9)

**Online resource 8.** Cluster validation


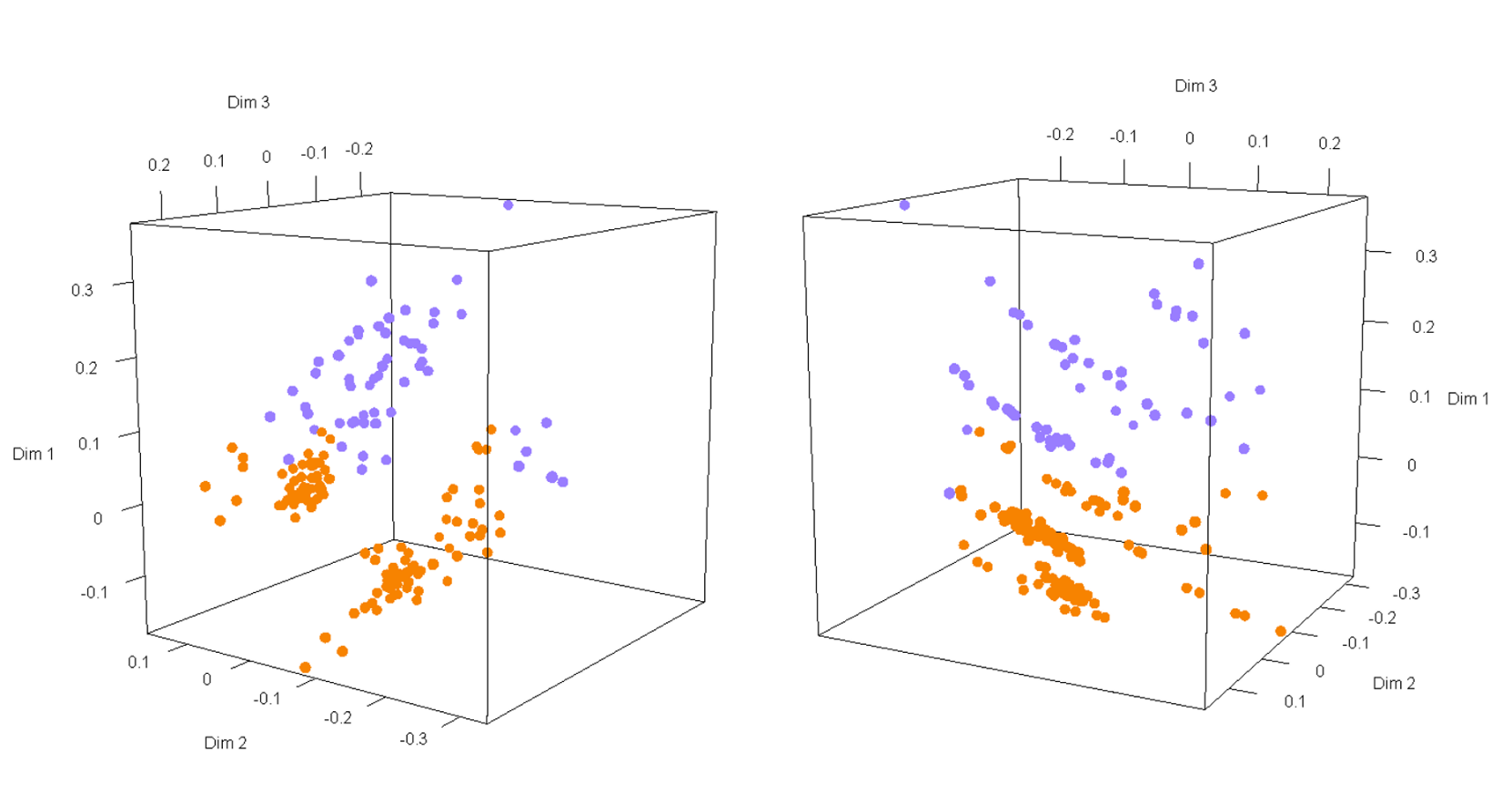
Figure A. Visualization of the clustering using a multidimensional scaling plot from two different views.

Figure B. Plot of the optimal number of clusters based on average silhouette width


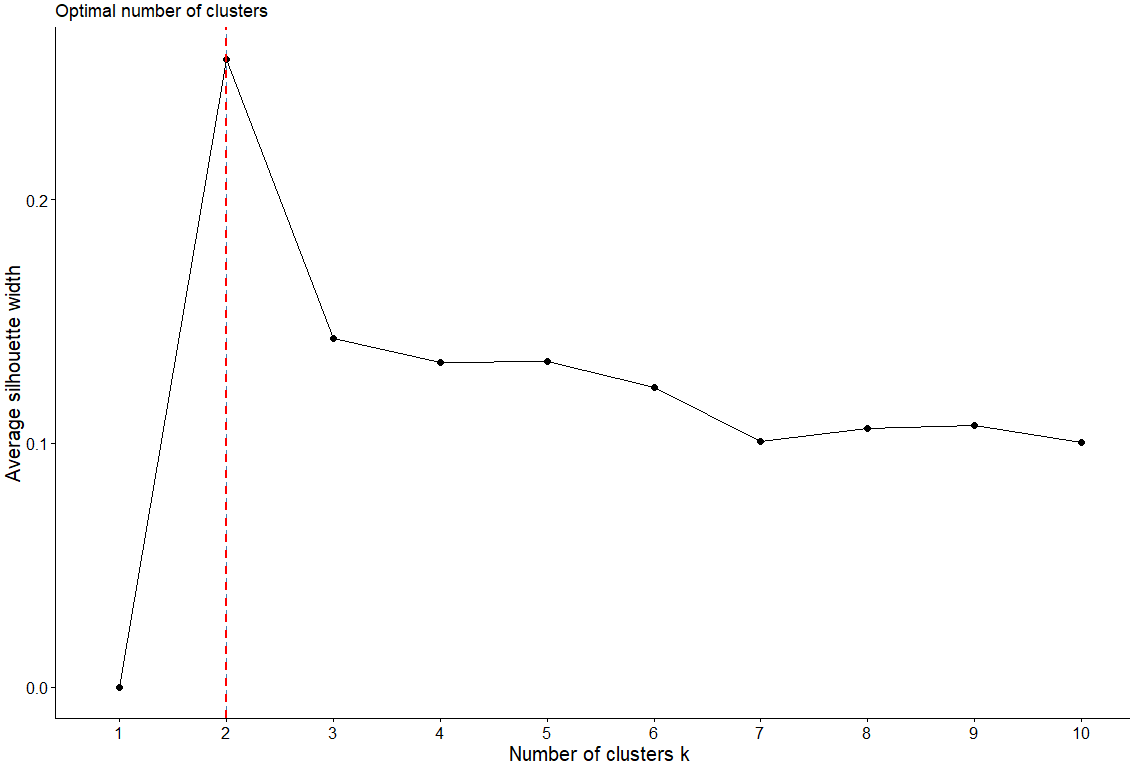
Figure C. Plot of the average silhouette width of the clusters


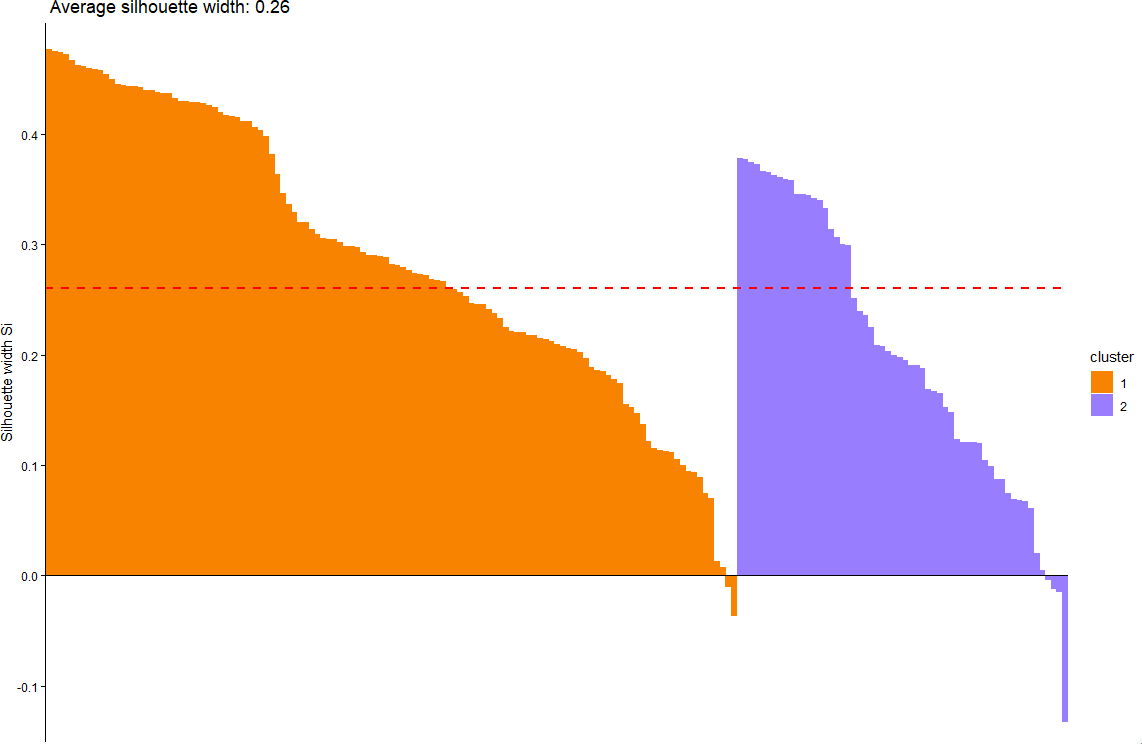

Supplement: Supplementary file 8 — (DOCX 211 KB) [file 431_2026_6973_MOESM8_ESM.docx]
