# Supplementary material for: Clusters in craniofacial microsomia and microtia according to facial morphology and craniofacial anomalies
Source: Eur J Pediatr. 2026 Apr 24;185(5):298. doi: 10.1007/s00431-026-06973-9 (PMC13109105; doi:10.1007/s00431-026-06973-9)
Supplement: Supplementary file 9 — (DOCX 309 KB) [file 431_2026_6973_MOESM9_ESM.docx]

**Online Resource 9** Asymmetry index (ASI) and facial symmetry (FS) scores for patients with and without velopharyngeal dysfunction (VPD).

| **Groups** | **VPD** | **1**  **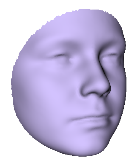** | **2**  **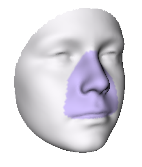** | **3**  **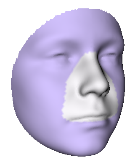** | **4**  **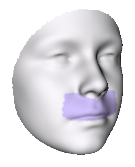** | **5**  **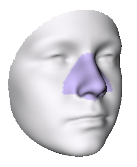** | **6**  **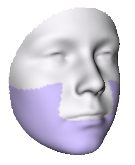** | **7**  **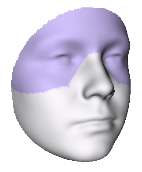** | **8**  **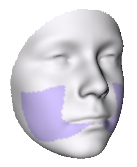** | **9**  **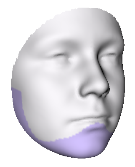** | **10**  **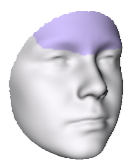** | **11**  **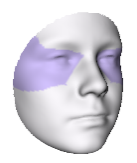** |
| --- | --- | --- | --- | --- | --- | --- | --- | --- | --- | --- | --- | --- |
| ***ASI^1^****, all* | **Yes,** n=21 | 1.87 | 1.22*** | 2.02 | 1.50** | 1.05** | 2.30 | 1.46 ◦ | 1.48 | 2.17 | 1.11 | 1.43* |
|  | IQR | [1.23-2.18] | [1.10-1.94] | [1.11-2.15] | [0.88-1.84] | [0.78-1.96] | [1.19-2.61] | [0.93-1.87] | [0.96-2.37] | [1.25-2.72] | [0.69-1.63] | [0.97-1.80] |
|  | **No**, n=139 | 1.36 | 0.89 | 1.44 | 0.88 | 0.75 | 1.53 | 1.15 | 1.38 | 1.71 | 1.05 | 1.19 |
|  | IQR | [1.00-1.96] | [0.65-1.22] | [1.03-2.10] | [0.62-1.41] | [0.55-1.06] | [0.99-2.46] | [0.82-1.47] | [0.84-2.01] | [1.00-2.78] | [0.70-1.48] | [0.78-1.51] |
| ***ASI^1^****, CM* | **Yes,** n=7 | 1.84 | 1.22 | 1.93 | 1.65 | 1.05 | 1.86 | 1.55 | 1.89 | 1.98 | 1.11 | 1.75 |
|  | IQR | [1.35-2.47] | [1.14-2.54] | [1.38-2.36] | [1.30-3.20] | [0.90-1.70] | [1.61-2.69] | [1.00-2.11] | [1.19-3.04] | [1.70-2.45] | [0.76-2.15] | [1.12-2.09] |
|  | **No**, n=131 | 1.35 | 0.88 | 1.44 | 0.88 | 0.75 | 1.52 | 1.15 | 1.32 | 1.67 | 1.05 | 1.17 |
|  | IQR | [1.00-1.92] | [0.64-1.21] | [1.02-2.07] | [0.62-1.33] | [0.55-1.05] | [0.98-2.43] | [0.81-1.44] | [0.83-2.00] | [1.00-2.75] | [0.70-1.44] | [0.78-1.46] |
| ***ASI^1^,***  *no cleft* | **Yes,** n=5 | 1.41 | 1.15 | 1.45 | 1.34 | 1.00 | 1.78 | 1.13 | 1.89 | 1.72 | 0.83 | 1.27 |
|  | IQR | [1.28-2.39] | [1.13-1.22] | [1.31-2.15] | [1.26-1.84] | [0.80-1.44] | [1.44-2.53] | [0.88-1.55] | [1.00-3.01] | [1.69-1.98] | [0.69-1.11] | [0.97-1.75] |
|  | **No**, n=107 | 1.23 | 0.80 | 1.30 | 0.78 | 0.73 | 1.35 | 1.09 | 1.18 | 1.48 | 0.98 | 1.07 |
|  | IQR | [0.94-1.68] | [0.60-1.05] | [0.94-1.82] | [0.57-1.11] | [0.54-1.05] | [0.86-2.19] | [0.75-1.40] | [0.76-1.68] | [0.91-2.25] | [0.60-1.38] | [0.78-1.44] |
| ***FS,*** *all* | **Yes,** n=26 | 1.57 | 1.41** | 1.60 | 1.42** | 1.26* | 1.54 | 1.34 | 1.38 | 1.60 | 1.19 | 1.30 |
|  | IQR | [1.15-1.92] | [1.20-1.84] | [1.08-1.78] | [1.16-1.99] | [1.01-1.73] | [1.05-1.94] | [1.12-1.62] | [0.92-1.84] | [1.25-2.07] | [0.94-1.96] | [0.99-1.58] |
|  | **No**, n=153 | 1.35 | 1.13 | 1.38 | 1.08 | 1.10 | 1.42 | 1.29 | 1.19 | 1.48 | 1.16 | 1.21 |
|  | IQR | [1.13-1.61] | [0.90-1.43] | [1.14-1.67] | [0.86-1.49] | [0.86-1.41] | [1.10-1.85] | [0.97-1.56] | [0.93-1.59] | [1.13-2.06] | [0.83-1.60] | [0.97-1.45] |
| ***FS,*** *CM* | **Yes,** n=8 | 1.49 | 1.36◦ | 1.54 | 1.48◦ | 1.34 | 1.72 | 1.28 | 1.51 | 1.64 | 0.87 | 1.38 |
|  | IQR | [1.07-1.96] | [1.25-1.95] | [0.92-1.86] | [1.25-2.23] | [1.11-1.78] | [1.06-2.20] | [0.71-1.33] | [0.96-2.31] | [1.22-2.41] | [0.74-1.07] | [0.70-1.49] |
|  | **No**, n=140 | 1.34 | 1.07 | 1.36 | 1.07 | 1.05 | 1.37 | 1.29 | 1.15 | 1.42 | 1.14 | 1.20 |
|  | IQR | [1.11-1.59] | [0.90-1.39] | [1.13-1.64] | [0.82-1.48] | [0.86-1.36] | [1.09-1.82] | [0.97-1.55] | [0.93-1.56] | [1.12-1.93] | [0.82-1.60] | [0.96-1.45] |
| ***FS****,*  *no cleft* | **Yes,** n=6 | 1.21 | 1.36 | 1.15 | 1.35 | 1.34 | 1.25 | 1.00 | 1.18 | 1.36 | 0.84 | 1.02 |
|  | IQR | [1.02-1.58] | [1.24-1.74] | [0.88-1.63] | [1.14-2.23] | [1.16-1.56] | [1.01-1.87] | [0.69-1.28] | [0.92-1.58] | [1.06-1.75] | [0.72-1.00] | [0.68-1.41] |
|  | **No**, n=116 | 1.30 | 1.07 | 1.31 | 1.07 | 1.04 | 1.26 | 1.23 | 1.08 | 1.33 | 1.14 | 1.18 |
|  | IQR | [1.08-1.50] | [0.91-1.38] | [1.07-1.55] | [0.86-1.49] | [0.82-1.34] | [1.05-1.71] | [0.94-1.55] | [0.87-1.44] | [1.06-1.78] | [0.81-1.59] | [0.93-1.45] |
| CM: excluding clefting, except for patients with macrostomia. | | | | | | | | | | | | |
| Values presented as median (interquartile range) | | | | | | | | | | | | |
| ^1^Unilateral patients only. | | | | | | | | | | | | |
| ***p<0.001, **p<0.01, *p<0.05, ◦ p≤0.1, Mann Whitney U test. Values for CM and no cleft subgroups are adjusted for multiple testing. | | | | | | | | | | | | |
